# CDC20 Is Regulated by the Histone Methyltransferase, KMT5A, in Castration-Resistant Prostate Cancer

**DOI:** 10.3390/cancers15143597

**Published:** 2023-07-13

**Authors:** Zainab A. H. Alebady, Mahsa Azizyan, Sirintra Nakjang, Emma Lishman-Walker, Dhuha Al-Kharaif, Scott Walker, Hui Xian Choo, Rebecca Garnham, Emma Scott, Katya L. Johnson, Craig N. Robson, Kelly Coffey

**Affiliations:** 1 Biosciences Institute, Newcastle Cancer Centre, Newcastle University, Newcastle upon Tyne NE2 4HH, UK; 2 Department of Laboratory and Clinical Science, College of Pharmacy, University of AL-Qadisiyah, Al-Diwaniya 58002, Iraq; 3 Bioinformatics Support Unit, Newcastle University, Newcastle NE2 4HH, UK; 4 Medical Laboratory Technology Department, College of Health Sciences, Public Authority of Applied Education and Training, Safat 13092, Kuwait; 5 School of Medicine, Newcastle University, Newcastle upon Tyne NE2 4HH, UK; 6 Translational and Clinical Research Institute, Newcastle Cancer Centre, Newcastle University, Newcastle upon Tyne NE2 4HH, UK

**Keywords:** CDC20, biomarker, KMT5A, p53, prostate cancer

## Abstract

**Simple Summary:**

The methyltransferase KMT5A is suggested as an oncogene in prostate cancer but the mechanisms underlying its oncogenic properties are poorly understood. This study uncovers genes and cellular pathways which are regulated by KMT5A in prostate cancer to obtain a better understanding of whether or not therapeutic targeting is viable. In particular, we focus on the key cell cycle protein, CDC20, which we reveal to be a KMT5A-regulated gene via two mechanisms; 1. the methylation of histone H4K20 within the *CDC20* promoter to enhance *CDC20* transcription and 2. the inhibition of p53 via direct methylation to release *CDC20* transcriptional repression. Furthermore, we demonstrate that *KMT5A* and *CDC20* are positively correlated in clinical samples of prostate cancer. Due to the roles that KMT5A and CDC20 play in cell cycle regulation and DNA repair processes, we propose that targeting the methylation activity of KMT5A will provide therapeutic benefits where these two oncogenic proteins are overexpressed.

**Abstract:**

The methyltransferase KMT5A has been proposed as an oncogene in prostate cancer and therefore represents a putative therapeutic target. To confirm this hypothesis, we have performed a microarray study on a prostate cancer cell line model of androgen independence following KMT5A knockdown in the presence of the transcriptionally active androgen receptor (AR) to understand which genes and cellular processes are regulated by KMT5A in the presence of an active AR. We observed that 301 genes were down-regulated whilst 408 were up-regulated when KMT5A expression was reduced. KEGG pathway and gene ontology analysis revealed that apoptosis and DNA damage signalling were up-regulated in response to KMT5A knockdown whilst protein folding and RNA splicing were down-regulated. Under these conditions, the top non-AR regulated gene was found to be CDC20, a key regulator of the spindle assembly checkpoint with an oncogenic role in several cancer types. Further investigation revealed that KMT5A regulates CDC20 in a methyltransferase-dependent manner to modulate histone H4K20 methylation within its promoter region and indirectly via the p53 signalling pathway. A positive correlation between KMT5A and CDC20 expression was also observed in clinical prostate cancer samples, further supporting this association. Therefore, we conclude that KMT5A is a valid therapeutic target for the treatment of prostate cancer and CDC20 could potentially be utilised as a biomarker for effective therapeutic targeting.

## 1. Introduction

Prostate cancer is the most common cancer in men in the UK. Whilst androgen receptor (AR)-targeting therapies have yielded significant patient benefits, relapse to treatment is a significant clinical problem. Hence, there is an urgent need to develop alternative therapeutics to treat advanced disease. The lysine methyltransferase, KMT5A, plays an oncogenic role in a number of cancers [1,2,3]. Indeed, *KMT5A* siRNA-mediated knockdown inhibits prostate cancer cell proliferation and KMT5A has been identified as an AR-interacting protein that is required for the transcription of the AR-regulated gene, *prostate specific-antigen (PSA)*, via the promotion of mono-methylation on histone H4 at lysine 20 (H4K20Me1) at the *PSA* promoter [4]. Furthermore, KMT5A plays a role in the epithelial–mesenchymal transition (EMT) and enhances the invasiveness of prostate cancer cell line models, independent of the AR through its interplay with ZEB1 [5]. Initially identified as the sole methyltransferase responsible for H4K20Me1, KMT5A was subsequently shown to methylate numerous other non-histone proteins, including p53 [6]. A greater understanding of KMT5A in the context of prostate cancer is required to determine whether or not it is a bona fide therapeutic target.

KMT5A activity is regulated via post-translational mechanisms during specific phases of the cell cycle. During the late S phase and at the G2/M transition, the levels of KMT5A are at their peak and found localised to mitotic chromosomes. As the cell moves through prophase to anaphase, KMT5A is phosphorylated at serine 29 by cdk1/cyclin B. This results in KMT5A dissociation from chromatin and stabilisation via the inhibition of KMT5A association with the APC^cdh1^ E3 ubiquitin ligase [7]. During anaphase, KMT5A is dephosphorylated by cdc14a/b, which in turn permits protein turnover to reduce KMT5A protein levels at G1. During G1, KMT5A levels are sustained, however, during the G1/S transition, SCF^skp2^ ubiquitin ligase targets KMT5A for protein turnover resulting in undetectable KMT5A protein. Interestingly, KMT5A interacts with the proliferating cell nuclear antigen (PCNA) at DNA replication foci and is essential for correct DNA replication [8] suggesting a high turnover rate of chromatin bound KMT5A by CRL4^cdt2^ [7]. The alterations in the levels of KMT5A throughout the cell cycle are mirrored by H4K20Me1 levels suggesting that methyltransferase activity is predominantly regulated by cellular KMT5A levels.

Cell cycle division 20 homologue (CDC20) is a cell cycle regulatory protein implicated in the spindle assembly checkpoint (SAC) and is required for cells to progress through mitosis. Specifically, CDC20 functions as a substrate recognition molecule and activator of APC to result in the ubiquitin-mediated turnover of its substrates. In particular, APC^CDC20^ functions during metaphase to anaphase to result in the destruction of cyclin B and securin, thereby allowing sister chromatids to segregate. CDC20 activity is inhibited by the mitotic checkpoint complex (MCC) and is only released to target its substrates once microtubule binding to the kinetochore and appropriate tension is achieved, thereby preventing genomic instability. Interestingly, there are suggestions that CDC20 may play a role in the DNA damage repair pathway via RAP80 [9] and REV1 [10] down-regulation. Furthermore, DNA damage-induced p53 can directly inhibit the expression of *CDC20* by associating with the *CDC20* promoter region and causing chromatin remodelling [11]. In addition, p21 can inhibit *CDC20* mRNA by associating with CDE-CHR elements in the *CDC20* promoter [12]. The depletion of *PHF8*, an H4K20Me1 demethylase, results in prolonged G2 and defective mitosis and it is itself a substrate of APC^CDC20^ [13] further suggesting that chromatin remodelling can be influenced by CDC20 levels.

CDC20 has been proposed to exhibit an oncogenic role in a number of cancers including prostate cancer [14]. Indeed, biochemical recurrence-free survival is lower in patients with high levels of *CDC20* compared to patients with low *CDC20* expression [15]. CDC20 itself is a target for ubiquitination by the E3 ligase SPOP, which is commonly mutated and non-functional in prostate cancers, providing an explanation for elevated CDC20 levels [16]. Furthermore, *CDC20* expression is associated with resistance to docetaxel [16,17] and is implicated in the wnt/Beta-catenin pathway which is oncogenic in advanced prostate cancer [17,18].

The aim of this study was two-fold; the first aim was to use pathway analysis to provide further evidence that KMT5A regulates oncogenic pathways and is a valid therapeutic target in prostate cancer and the second was to identify individual genes that are regulated by KMT5A in a model of castration-resistant prostate cancer as potential biomarkers for KMT5A activity. Indeed, we show that a number of oncogenic pathways are down-regulated upon *KMT5A* knockdown and we identified and validated *CDC20* as a KMT5A-regulated gene.

## 2. Materials and Methods

### 2.1. Antibodies

Antibodies used in this study included KMT5A (cell signalling), CDC20 (Ab190711, and AbCam), PARP1/2 (clone H250, sc-7150, Santa Cruz Biotechnology, Dallas, TX, USA), MDM2 (Clone N-20, sc-813, Santa Cruz Biotechnology), p21 (ab-4, Calbiochem), p53 (pAb-421#OP03, Calbiochem), p53-S15-P (cell signalling), p53-K382-Ac (ab75754, AbCam), H4K20Me1 (Ab9051, AbCam), H4 (07-108, Merck, Darmstadt, Germany), anti-phospho-histone H2AX (Ser139) (clone JBW301, Millipore Corp., Burlington, MA, USA) α-tubulin (clone DM1A, T9026, Sigma, St. Louis, MO, USA), and GAPDH (clone 1E6D9, Proteintech, Rosemont, IL, USA).

### 2.2. Compounds

Dihydrotestosterone (DHT) (Sigma) was prepared in ethanol at a final concentration of 10 mM and stored at −80 °C. KMT5A inhibitors UNC0379 (S7570, Selleckchem, Houston, TX, USA) and Ryuvidine (2609, R&D Systems, Minneapolis, MN, USA) were purchased in powder form and resuspended in DMSO to a final concentration of 50 mM and 20 mM, respectively. Solutions were stored at −80 °C for no longer than 1 month. Nutlin 3 was provided by Prof. John Lunec (Newcastle Cancer Centre).

### 2.3. Cell Culture

LNCaP cells, a model of androgen dependence, and AR negative PC3 cells were purchased from American Type Culture Collection (Manassas, VA, USA); LNCaP-AI cells, a model of androgen independence, were generated in-house as described previously [19]. Cells were maintained as previously described [20].

Short tandem repeat profiling was used to authenticate the cell lines used in this study (NewGene, Newcastle upon Tyne, UK). MycoAlert (Lonza, UK) was used to routinely test for the presence of mycoplasma.

### 2.4. siRNA

The reverse transfection of cell lines with siRNA sequences (25 nM) was carried out using Lipofectamine RNAiMAX (Invitrogen) in accordance with the manufacturer’s protocol. Either qPCR or Western blotting confirmed successful knockdown. Non-silencing (N/S): UUCUCCGAACGUGUCACGU[dT][dT]; siKMT5A_1: CCAUGAAGUCCGAGGAACA[dT][dT]; siKMT5A_2: GATGCAACTAGAGAGACA[dT][dT]; siCDC20_1 CGGAAGACCUGCCGUUACA[dT][dT]; siCDC20_2: GGGCCGAACUCCUGGCAAA[dT][dT].

### 2.5. Western Blotting and Quantitative Polymerase Chain Reaction

Western [21] and qPCR analysis [20] were performed as described previously. Primer sequences are detailed in Supplementary Table S1.

### 2.6. Microarray

Cellular RNA was extracted using Trizol^®^ (Invitrogen, Waltham, MA, USA) and quality-checked using Agilent Bioanalyzer 2100 prior to analysis using Illumina HT-12 v4.0 Expression BeadChip (Oxford Genomics Centre, The Wellcome Trust Centre for Human Genetics, University of Oxford, Oxford, UK).

R package ‘Lumi’ was used for array processing, background correction, normalisation and quality control checks. Variance-stabilising transformation was used to convert probe intensity values to VSD (variance-stabilised data). The array normalisation method used was the robust spline normalisation (RSN) method. Outlier samples, poor quality probes (detection threshold < 0.01) and probes that were not detected were removed from downstream analysis. R package ‘Limma’ was then used to perform a differential expression analysis with *p*-values adjusted using the Benjamini–Hochberg method [22] to take into account the false discovery rate (FDR). Analysis was performed by the Bioinformatics Support Unit (Newcastle University).

Data can be found at GSE233350.

### 2.7. RNA-Seq Analysis

Fastq files were downloaded from NCBI GEO (GSE211638, [23]), and RNA-STAR [24] analysis was performed to align raw reads to genome build GRCh37/hg19; QC checks were performed with FastQC. Gene counts were generated using ht-seq count [25] and Gencode v19. Differential expression analysis was carried out using the DESeq2 [26] package (R/Bioconductor) to compare the vehicle versus 10 nM DHT-treated samples.

### 2.8. Chromatin Immunoprecipitation Assays

LNCaP-AI and LNCaP cells were reverse-transfected with either 25 nM N/S or a pool of 2 *KMT5A*-targeting siRNAs for 72 h in steroid-depleted media followed by chromatin immunoprecipitation as described by Schmidt et al. [27].

For immunoprecipitations, 2 μg of H4K20Me1 (Ab9051, AbCam) or 2 μg of a non-specific isotype control (DAKO) was used. qPCR analysis of immunoprecipitated DNA was performed using primers specific to the *CDC20* promoter (Fwd: 5′-CCGCTAGACTCTCGTGATAGC-3′; Rev: 5′-TGGCTCCTTCAAAATCCAAC-3′) as previously described [28]. The average fold difference of the % input between experimental arms for at least three independent experiments is presented.

### 2.9. Sulforhodamine B Growth Analysis

Cellular growth was assessed as described [21].

### 2.10. Gamma H2AX Assay

Knockdown was carried out over 72 h in LNCaP-AI and LNCaP cells using either N/S or *KMT5A*-targeting siRNA. Cells were harvested and stained for phospho-histone H2AX (Ser139) as previously described [21].

## 3. Results

### 3.1. Identification of KMT5A-Regulated Genes in Androgen-Independent Prostate Cancer

KMT5A has been proposed as a therapeutic target in prostate cancer; however, in this context, KMT5A is still largely understudied. Indeed, no study has identified which genes KMT5A can regulate in castration-resistant prostate cancer. To this end, *KMT5A* mRNA was knocked down using two independent siRNA sequences in the LNCaP-AI cell line model of androgen independence. After 72 h of knockdown under steroid-depleted conditions, the androgen, DHT (10 nM), was applied for 24 h prior to RNA isolation and analysis using an Illumina Human HT-12 microarray.

**Figure 1:**
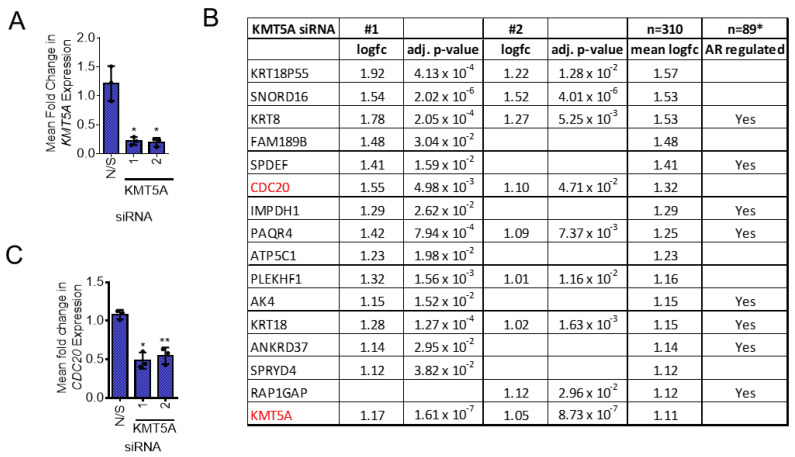
KMT5A-regulated genes in LNCaP-AI cells. (**A**) LNCaP-AI cells reverse-transfected with 25 nM siRNA targeting *KMT5A* or a non-silencing control (N/S) in steroid-depleted media. After 72 h, 10 nM DHT was added to the cells for a further 24 h. RNA was isolated, quality-checked, and its gene expression profiles determined using Illumina HT-12 v4.0 Expression BeadChip Microarray. Three independent experimental repeats were performed. Data analysis confirmed successful *KMT5A* knockdown. (**B**) Table of genes ranked for their down-regulation in response to *KMT5A* knockdown in the presence of DHT stimulation. Genes which were down-regulated more than *KMT5A* are shown (full gene lists can be found in the Supplementary Information). *GSE211638 [23]. (**C**) *CDC20* expression levels in response to *KMT5A* knockdown as determined via microarray analysis. One-way ANOVA with Dunnett’s multiple comparisons test. * *p* < 0.05; ** *p* < 0.01.

The significant knockdown of *KMT5A*, with both siRNAs, was confirmed within the microarray data set as >80% prior to further analysis (Figure 1A). In the presence of an active AR found after stimulation with DHT for 24 h, we found 408 genes up-regulated and 310 genes down-regulated. (Supplementary Tables S2 and S3). Of these genes, 29% have previously been shown to be AR-regulated in LNCaP cells (Supplementary Tables S2 and S3) [23]. In order to understand which cellular pathways and biological processes are affected under these conditions, the gene lists generated were used in KEGG pathway analysis and gene ontology analysis using DAVID [29,30]. We did observe a level of inconsistency between the siRNA oligos even though the level of *KMT5A* knockdown was consistent (Figure 1A), a common issue when using siRNAs to assess multiple gene expressions, highlighting the importance of further validation studies on any target identified. Hence, all genes which showed a statistically significant change irrespective of the siRNA sequence were included in gene lists for this analysis to enhance confidence in the pathways and genes identified. This analysis revealed the significant up-regulation of PI3K-Akt signalling, apoptosis, p53 signalling and signal transduction whilst the pathways found to be significantly down-regulated included splicing, protein folding, cell division and transcriptional regulation (Supplementary Tables S4–S7). Taken together, the cellular processes and genes altered in this analysis further support our hypothesis that KMT5A is a potential therapeutic target for prostate cancer.

In terms of individual genes which were down-regulated in response to *KMT5A* knockdown, *CDC20* was identified as the sixth most down-regulated gene after AR-regulated genes such as *KRT8* and *SPDEF* (Figure 1B,C). Due to its role in the cell cycle and previous characterisation as an oncogene [14,17], this gene was chosen for further study as a potential pharmacodynamic biomarker for KMT5A therapeutic targeting and a KMT5A effector protein.

### 3.2. KMT5A Depletion Reduces CDC20 Expression

**Figure 2:**
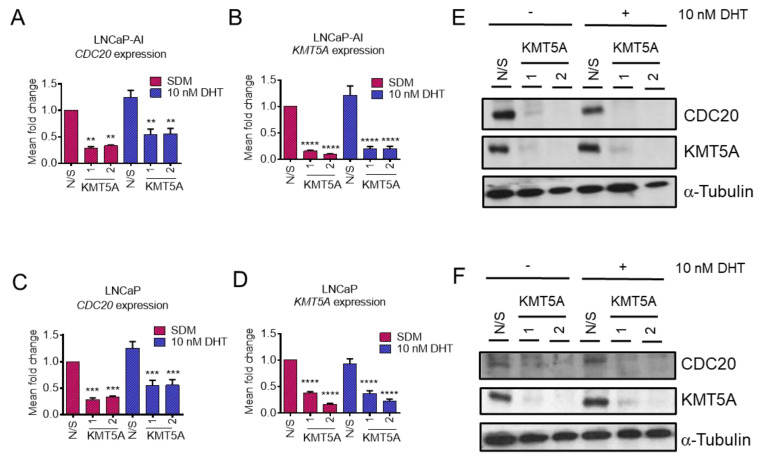
*CDC20* is a KMT5A-regulated gene. (**A**) LNCaP-AI cells were reverse transfected with 25 nM siRNAs targeting *KMT5A* or a non-silencing control (N/S) in steroid-depleted media. After 72 h, 10 nM DHT or a vehicle control was added to the cells for a further 24 h. RNA was isolated and *CDC20* mRNA and (**B**) *KMT5A* mRNA were quantified via qPCR. The same experiment was performed in (**C**) LNCaP cells, and *CDC20* levels and (**D**) *KMT5A* knockdown were confirmed via qPCR. Data are expressed as the mean fold change over 3 independent experiments, +/− SEM. (**E**) Determination of the protein levels of CDC20 and KMT5A via Western blotting in LNCaP-AI and (**F**) LNCaP cells. Alpha-tubulin was used as a loading control. Data shown are representative of 3 independent experiments. Two-way ANOVA with Dunnett’s multiple comparisons test; ** *p* < 0.01; *** *p* < 0.001; **** *p* < 0.001. The uncropped blots are shown in File S1.

In order to validate *CDC20* as a KMT5A-regulated gene, further experiments were conducted in both LNCaP-AI cells and the parental, androgen-sensitive LNCaP cell line. *KMT5A*-targeting siRNAs were transfected into both cell lines in steroid-depleted media for 72 h prior to stimulation with 10 nM DHT or the vehicle for a further 24 h. qPCR confirmed a significant reduction in the expression of *CDC20* in LNCaP-AI cells (Figure 2A) when *KMT5A* was knocked down (*p* < 0.01) (Figure 2B), which is consistent with our microarray data (Figure 1C). In addition, parental LNCaP cells also exhibited a significant reduction in *CDC20* expression (Figure 2C) irrespective of DHT stimulation upon significant *KMT5A* knockdown (*p* < 0.001) (Figure 2D). Furthermore, a robust reduction in CDC20 protein levels was consistently observed in both cell lines (Figure 2E,F) confirming that CDC20 is regulated by KMT5A, at the level of transcription, in both cell lines irrespective of AR activation.

### 3.3. CDC20 Depletion Does Not Enhance KMT5A Protein Expression

**Figure 3:**
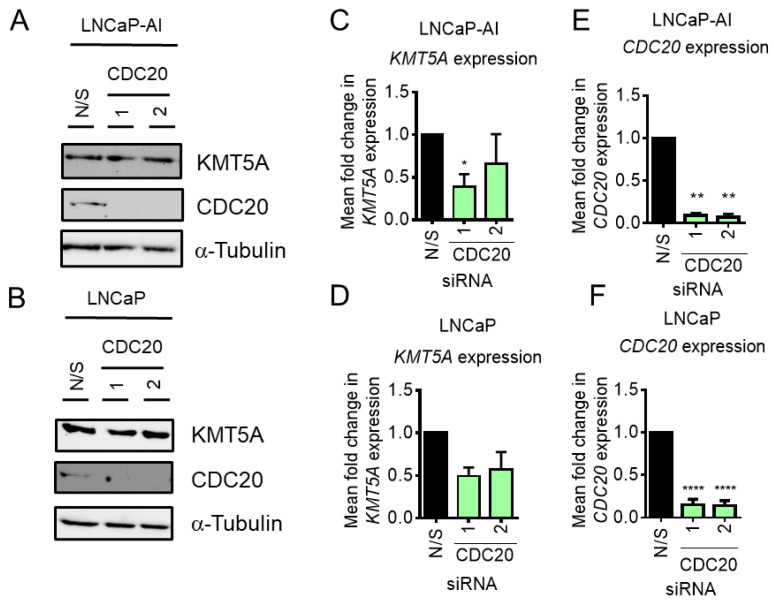
*CDC20* knockdown does not affect KMT5A protein levels. (**A**) LNCaP-AI cells and (**B**) LNCaP cells reverse-transfected with 25 nM siRNA targeting *CDC20* or a non-silencing control (N/S) for 72 h prior to analysis via Western blot analysis. Alpha-tubulin was used as a loading control. (**C**) LNCaP-AI and (**D**) LNCaP cells reverse-transfected with 25 nM siRNA targeting *CDC20* or a non-silencing control (N/S) for 72 h prior to RNA isolation and qPCR analysis for *KMT5A* and (**E**,**F**) *CDC20* mRNA levels. *HPRT1* was used as a housekeeping gene. Data are expressed as mean fold change +/− SEM. One-way ANOVA was used to determine statistical significance. * *p* < 0.05; ** *p* < 0.01; **** *p* < 0.0001. The uncropped blots are shown in File S1.

KMT5A is phosphorylated to protect it from ubiquitin-mediated degradation by APC^cdh1^ during late mitosis [7]. In addition, due to the similarity in recognition mechanisms between CDH1 and CDC20 for targeting proteins to the APC complex, it was suggested that CDC20 may also bind and recognise KMT5A in the absence of phosphorylation [7]. This raised the question of whether or not a feedback mechanism exists between these two proteins to help maintain correct cell cycle progression. To test this theory, *CDC20* was knocked down in our cell line models and KMT5A levels were assessed at both the transcript and protein level. Interestingly, when KMT5A protein levels were examined subsequent to *CDC20* knockdown, no change was observed (Figure 3A,B), suggesting that KMT5A protein turnover does not take place when CDC20 is present in the cell. However, a decrease in *KMT5A* transcripts by ~50% was observed in both cell lines (Figure 3C,D) upon the robust depletion of *CDC20* (Figure 3E,F) although this was not statistically significant. Therefore, it was concluded that CDC20 did not play a significant role in KMT5A protein regulation under our experimental conditions and that KMT5A sits upstream of CDC20.

### 3.4. KMT5A Expression Correlates with CDC20 Expression in Prostate Cancer Patients

**Figure 4:**
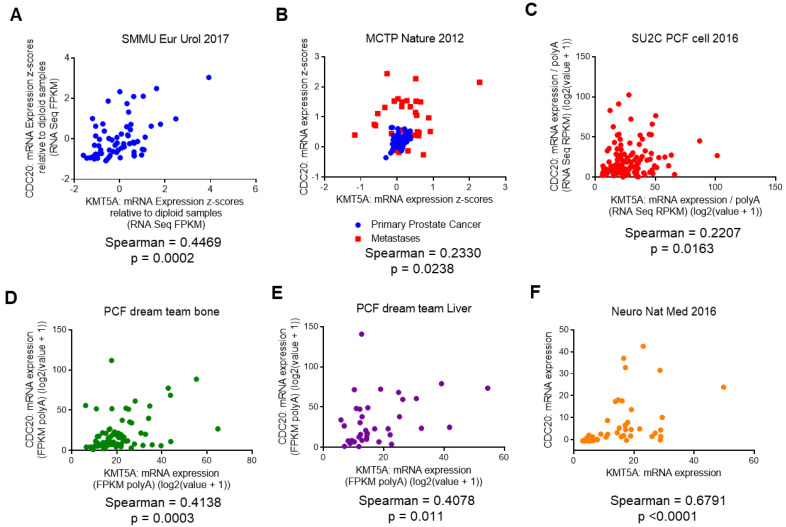
*KMT5A* and *CDC20* are positively correlated in clinical prostate cancer samples. Correlation in expression between *KMT5A* and *CDC20* was carried out in publicly available datasets in cBioportal. (**A**) Treatment naïve radical prostatectomies (n = 65) from [33] (**B**) Both primary and metastatic prostate cancer (n = 94) from [34] (**C**) Metastatic prostate adenocarcinoma samples (n = 150) from [35] (**D**) Bone metastatic prostate adenocarcinoma samples (n = 72/266) and (**E**) Liver metastatic prostate adenocarcinoma samples (n = 38) from [36] (**F**) Prostate neuroendocrine carcinoma samples (n = 49) from [37]. Spearman correlation (two-tailed) was calculated using Graphpad software v6.

To confirm whether or not our in vitro findings could be translated into clinical specimens of prostate cancer, we interrogated publicly available datasets to confirm a positive correlation between *CDC20* and *KMT5A* transcripts. Upon the interrogation of data sets available in cBioportal [31,32], we found a significant positive correlation between *KMT5A* and *CDC20* transcripts in a number of data sets. In a cohort of 65 treatment-naïve radical prostatectomies [33] a positive correlation between *CDC20* and *KMT5A* transcripts was observed (Spearman = 0.45; *p* = 0.0002) (Figure 4A). Similarly, in the MCTP dataset [34], a positive correlation was also observed (Spearman = 0.23; *p* = 0.024) (Figure 4B). However, this dataset contains samples from primary (blue) and metastatic prostate cancer (red). Upon the correlation analysis of these individual sample types, it was observed that the correlation between *CDC20* and *KMT5A* was strongest in the primary prostate samples (Spearman = 0.28; *p* = 0.033; n = 59) and no statistically significant correlation was observed in the metastatic samples (Spearman = 0.034; *p* = 0.85; n = 35), although the sample numbers were lower. However, in the metastatic cohort reported by Robinson et al. [35] a statistically significant correlation was observed between *CDC20* and *KMT5A* (Spearman = 0.22; *p* = 0.016; n = 118) (Figure 4C). Furthermore, significant correlations were observed in bone metastases (Spearman = 0.41; *p* = 0.0003; n = 72) (Figure 4D) and liver metastases (Spearman = 0.408; *p* = 0.011; n = 38) (Figure 4E) in the samples from Abida et al. [36]. Interestingly, the highest positive correlation between *CDC20* and *KMT5A* expression was seen in prostate neuroendocrine carcinoma samples (Spearman = 0.68; *p* < 0.0001; n = 49) (Figure 4F) [37]. Taken together, this suggests that the positive correlation between *KMT5A* and *CDC20* observed in our cell line models is also observed in advanced prostate cancer.

### 3.5. KMT5A Inhibition Reduces CDC20 Expression and Reduces Prostate Cancer Cell Proliferation

KMT5A plays a role in the cell cycle and as such, knockdown of *KMT5A* has been shown to inhibit cellular proliferation [38,39]. Indeed, we observed a reduction in proliferation upon *KMT5A* knockdown in both the cell line models used in this study (Supplementary Figure S1A–C). In particular, proliferation was most affected under steroid depleted conditions. Furthermore, as expected, knockdown of *CDC20* resulted in a robust and significant inhibition of cellular proliferation (Supplementary Figure S1D,E). Together this provides supporting evidence that both proteins play a role in prostate cancer cell proliferation.

**Figure 5:**
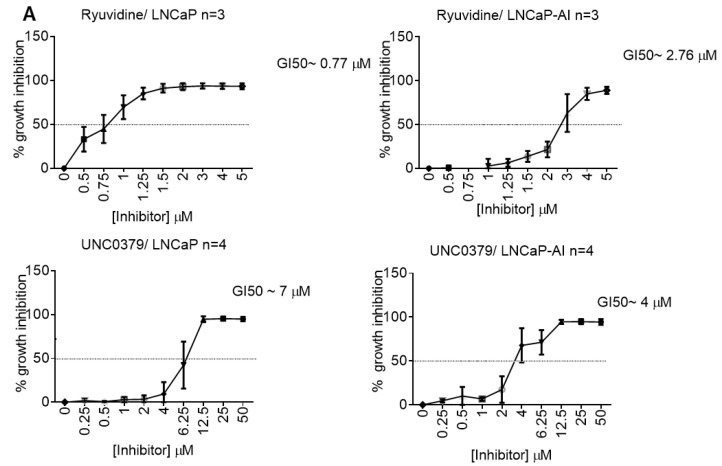

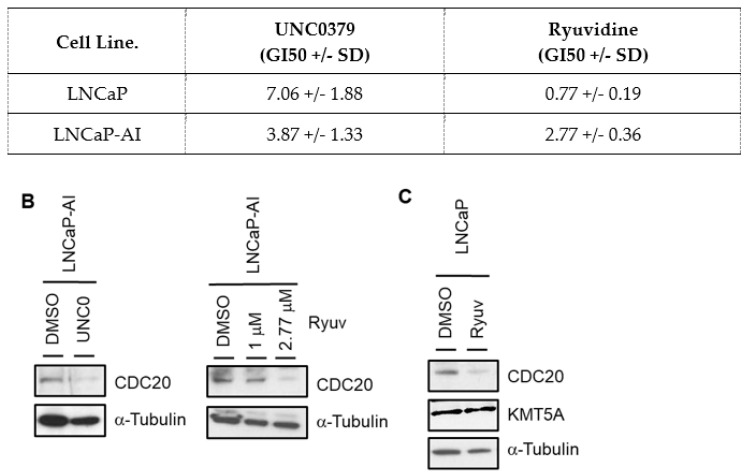
KMT5A inhibitors restrict prostate cancer cell growth and down-regulate CDC20. (**A**) LNCaP-AI and LNCaP cells treated with a dose range of UNC0379 or Ryuvidine and GI50 concentrations determined via SRB assay after 3 doubling times. Data are expressed as mean % growth inhibition +/− SEM from 3 independent experiments. Mean GI50 values are tabulated +/− SD. (**B**) LNCaP-AI cells treated with either UNC0379 (7 μM) or Ryuvidine (1 μM or 2.77 μM) for 48 h prior to protein analysis via Western blotting. (**C**) LNCaP cells were treated with Ryuvidine (0.7 μM) for 48 h prior to protein analysis via Western blotting. Data shown are representative of 3 independent experiments. The uncropped blots are shown in File S1.

In order to confirm that the methyltransferase activity of KMT5A is important for the regulation of *CDC20* expression in prostate cancer cell lines we used two molecules that have shown inhibitory activity against KMT5A, namely UNC0379 and Ryuvidine [40,41] (Supplementary Figure S2). Firstly, we determined the GI50 values for LNCaP and LNCaP-AI cells (Figure 5A). Interestingly, we found that Ryuvidine was a more potent inhibitor than UNC0379 in LNCaP cells, However, in LNCaP-AI cells there was not such a large difference in efficacy. Secondly, we used a titration of doses of both inhibitors and investigated the dose dependent effects on both KMT5A and its target histone mark, H4K20Me1. We observed that UNC0379 resulted in a robust decrease in H4K20Me1 levels in LNCaP-AI and a modest reduction in LNCaP cells when total H4 levels are taken into account. This coincided with a decrease in KMT5A protein levels in LNCaP cells whilst KMT5A levels showed minimal change in LNCaP-AI cells. Ryuvidine also demonstrated a dose dependent reduction in KMT5A activity in LNCaP cells whilst a decrease in H4K20Me1 was more difficult to achieve in LNCaP-AI cells thereby reflecting the sensitivity differences to Ryuvidine between these two cell lines (Supplementary Figures S2 and S3).

Using the GI50 concentrations for each drug in LNCaP-AI cells we investigated the levels of CDC20 by Western blotting, demonstrating that both drugs result in CDC20 reduction (Figure 5B). Furthermore, when the more potent Ryuvidine was used in LNCaP cells at the GI50 dose a reduction in CDC20 levels was observed (Figure 5C). Together, this led us to conclude that KMT5A enzymatic activity is important in the regulation of CDC20 protein levels.

### 3.6. KMT5A Knockdown Reduces H4K20Me1 at the CDC20 Promoter

**Figure 6:**
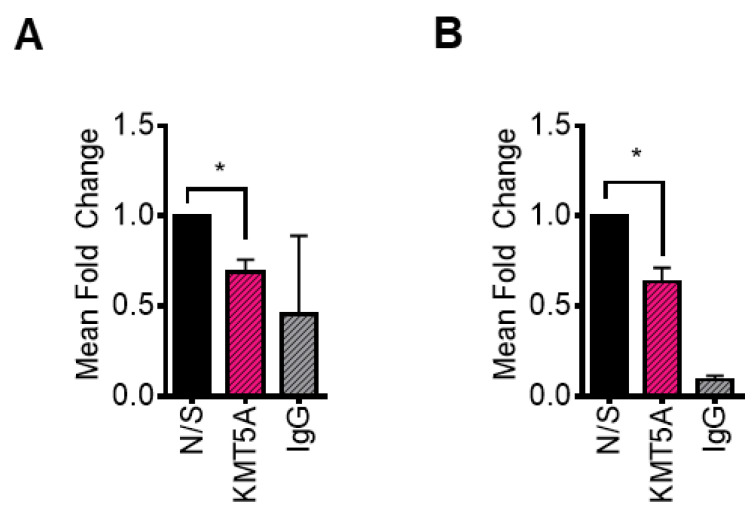
H4K20Me1 is reduced at the CDC20 promoter in response to KMT5A knockdown. *KMT5A* was knocked down in (**A**) LNCaP-AI and (**B**) LNCaP cells growing in steroid depleted media for 72 h. Chromatin was collected, and immunoprecipitation for was H4K20Me1 carried out. Isolated DNA was purified and primers targeting the *CDC20* promoter region were used to determine the levels of H4K20Me1 association with this region. Experiments were performed 3 times and data are expressed as the mean fold change relative to the non-silencing control, +/− SEM. IgG was used as a negative control. Student’s *t*-test * *p* < 0.05.

In order to confirm that KMT5A can directly regulate the expression of *CDC20* via the mono-methylation of its only histone target, H4K20, chromatin immunoprecipitation assays were performed in both LNCaP-AI and LNCaP cells subsequent to *KMT5A* knockdown using a siRNA pool of siKMT5A_1 and siKMT5A_2. Upon *KMT5A* knockdown in both cell lines growing in steroid depleted media, a significant reduction in H4K20Me1 was observed at the *CDC20* promoter region (Figure 6A,B). This led us to conclude that KMT5A can directly modulate the expression of *CDC20* via the methylation of H4K20 within the promoter region.

### 3.7. p53 Mediates KMT5A Regulation of CDC20 Expression

**Figure 7:**
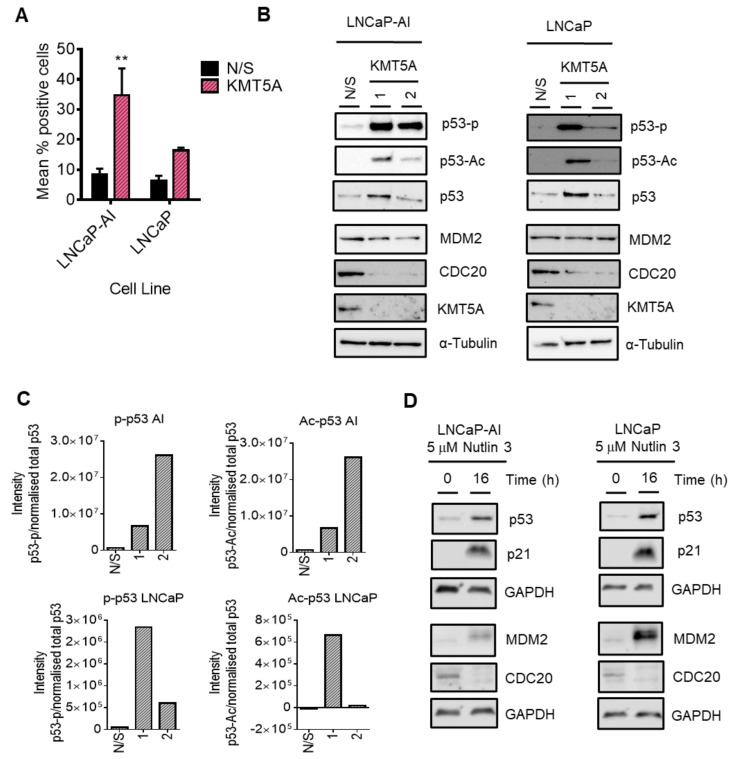
KMT5A regulates *CDC20* via p53 activation. (**A**) LNCaP-AI and LNCaP cells reverse transfected with either a pool of 3 *KMT5A*-targeting siRNAs or a non-silencing (N/S) control for 72 h prior to the assessment of γ-H2AX via flow cytometry. Scatter plots for experimental replicates can be found in Supplementary Figure S4. (**B**) LNCaP-AI and LNCaP cells reverse-transfected with 2 independent siRNA sequences for 72 h prior to Western blotting analysis for CDC20, p53, p-p53, p53-Ac, MDM2, and KMT5A. (**C**) Densitometry of Western blots shown in (**B**). The background was subtracted from the intensity values prior to intensity normalisation against an appropriate loading control. Intensities for post-translationally modified proteins were normalised to total protein intensity. Additional experimental repeats can be found in Supplementary Figure S6. (**D**) LNCaP-AI and LNCaP cells were treated with Nutlin 3 (5 μM) for 0 and 16 h prior to Western blotting analysis. Western blot data shown is representative of 3 independent experiments. Two-way ANOVA test was used to determine statistical significance for data shown in (**A**). ** *p* < 0.01 The uncropped blots are shown in File S1.

Whilst KMT5A methyltransferase activity is important in regulating the expression of *CDC20* via the regulation of H4K20Me1 within the *CDC20* promoter, KMT5A can also methylate non-histone proteins, including p53, to regulate functional activity. Interestingly, in our pathway analysis we uncovered some pathways within which CDC20 can be modulated. In particular, p53 directly down-regulates *CDC20* expression via association with its promoter in response to DNA damage [11]. Secondly, in the absence of DNA damage, p53 can regulate *CDC20* expression via a CDE-CHR element, independent of p21, when p53 is over-expressed [11,14,42]. To determine whether or not DNA damage in response to KMT5A knockdown was influencing this mechanism, we investigated the levels of γ-H2AX in both LNCaP-AI and LNCaP cells after *KMT5A* knockdown. Consistent with other reports [39], *KMT5A* knockdown resulted in an increased level of DNA damage as denoted by a robust ~3.5 fold and ~2 fold increase in γ-H2AX levels in LNCaP-AI cells and LNCaP cells, respectively (Figure 7A), further suggesting that p53 could be a mediator of KMT5A effects on *CDC20* levels. Indeed, KMT5A is well-known to methylate p53 at K382 to reduce p53 activation [6], and the down-regulation of KMT5A in response to DNA damage has been shown to result in the conversion of this mono-methylation state into a di/tri- methylation state on K382 to increase p53 stability [6,43]. Therefore, we hypothesised that knockdown of *KMT5A* would shift the equilibrium from mono-methylated p53 to acetylated p53, thereby resulting in p53 activation and the subsequent repression of *CDC20* expression. To test this theory, *KMT5A* was knocked down in both LNCaP-AI and LNCaP cells prior to Western blotting for changes in p53 post-translational modifications and MDM2. We observed no alterations in total p53 protein levels in either cell line with siKMT5A_2; however, p53 levels were increased with siKMT5A_1. Nonetheless, a robust increase in acetylation at K382 and an increase in p53-phosphorylation at serine 15 which is associated with enhanced DNA binding was still observed with both siRNA sequences (Figure 7B,C and Figure S6). To confirm that p53 activation results in the down-regulation of CDC20 protein levels, we treated both LNCaP-AI and LNCaP cells with the MDM2 inhibitor, Nutlin 3. In both cell lines, CDC20 protein levels were reduced when p53 was activated (Figure 7D). Taken together, this suggests that p53 activation via *KMT5A* knockdown results in the repression of *CDC20*.

### 3.8. CDC20 Is Down-Regulated by Protein Turnover in the Absence of p53

**Figure 8:**
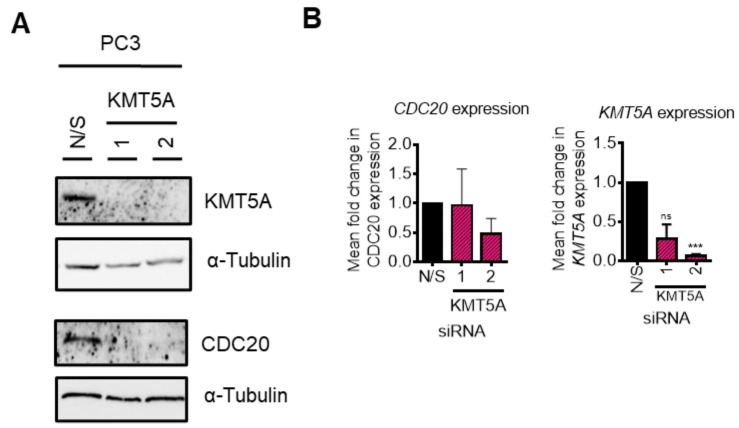
KMT5A regulates protein turnover in the absence of p53. (**A**) *KMT5A* was knocked down with 2 independent siRNAs for 72 h. Protein was collected and analysed via Western blotting for KMT5A and CDC20, and α-tubulin was used as a loading control. (**B**) Expression of *CDC20* and *KMT5A* in a parallel experiment analysed via qPCR. Data are shown as the mean fold change relative to that of N/S controls over 3 experimental repeats, +/− SEM. One-way ANOVA was used to determine statistical significance. *** *p* < 0.01; ns: not significant. The uncropped blots are shown in File S1.

As p53 signalling was found to be up-regulated in our KEGG pathway analysis (Supplementary Table S4), we questioned whether or not KMT5A was able to regulate *CDC20* if p53 was not present. As p53 loss is a common phenomenon in cancers, this raised questions regarding the applicability of CDC20 as a KMT5A biomarker for those patients whose tumours lack p53 expression. To investigate further, we performed *KMT5A* knockdowns in p53 null, PC3 cells and performed Western blotting and qPCR analyses of *CDC20* levels. Surprisingly, *KMT5A* knockdown was still able to robustly reduce CDC20 protein levels in this cell line (Figure 8A). However, *CDC20* mRNA levels were unaffected (Figure 8B) suggesting that CDC20 post-translational changes occurred causing alterations in protein turnover in the absence of p53. Therefore, if a protein biomarker read-out could be used then CDC20 may remain as a valid KMT5A activity biomarker.

## 4. Discussion

Alternative therapeutic targets are urgently required for the treatment of advanced prostate cancers which have relapsed after current standard-of-care therapies. As the androgen receptor remains a driver of disease in therapy relapse, proteins which positively modulate the transcriptional activity of the androgen receptor are proposed as putative therapeutic targets. The protein methyltransferase KMT5A has been shown to interact with the androgen receptor [4] and was proposed to offer therapeutic benefits to prostate cancer patients. However, the mechanisms by which KMT5A contributes to prostate cancer progression remains poorly understood.

We uncovered that KMT5A can regulate the levels of the cell cycle regulator protein CDC20 both directly at the chromatin level via the modulation of histone methylation, and indirectly via the methylation of the tumour suppressor protein, p53. This relationship between CDC20 and KMT5A is supported by a significant positive correlation between *KMT5A* and *CDC20* transcripts in prostate cancer patients (Figure 4). Whilst this relationship is independent of the androgen receptor (Figure 2 and Figure S5), both proteins are described as oncogenes in prostate cancer. Critically, there are no reports describing the methylation-specific regulation of CDC20.

KMT5A is the only known methyltransferase to monomethylate histone H4K20. As the H4K20Me1 mark is traditionally associated with a compact chromatin landscape and gene repression [44,45,46,47], it is counterintuitive that KMT5A should function to facilitate *CDC20* transcription. However, KMT5A-mediated H4K20Me1 is now well-documented to function as a transcriptional activator for some genes [48,49]. Where this has been observed, there is generally a transcription factor which is implicated, for example TWIST [48]. Furthermore, H4K20Me1 is associated with actively transcribing gene bodies [50] and more recently has been found to result in chromatin accessibility in highly transcribed genes throughout the cell cycle [51]. A role for KMT5A in the pause and release of RNA pol II has also been revealed [52], further supporting the complex role of KMT5A in the positive regulation of gene transcription.

The methylation-dependent regulation of p53 activity by KMT5A is key to ensuring transcriptional activation [6,43], further highlighting the ability of KMT5A to influence gene expression programmes at multiple levels. Consistently, we observed that knockdown of *KMT5A* resulted in enhanced p53 acetylation at K382, which can only occur if this residue is not methylated. Importantly, it is the subsequent phosphorylation event at S15 which facilitates the association of p53 with DNA which is enhanced upon *KMT5A* knockdown. This would permit the recruitment of HDAC1 and mSin3a to the *CDC20* promoter to allow chromatin remodelling to occur and thereby inhibit the transcription of *CDC20* [11]. Importantly, we observed slightly different effects with each KMT5A-targeting siRNA in this experiment with regard to the ability of KMT5A knockdown to stabilise p53, making the interpretation of p53 post-translational modifications more complex. However, densitometry confirmed that both p53 phosphorylation and acetylation do increase with KMT5A knockdown with both siRNA sequences (Figure 7C). Therefore, it appears that there are two complementary mechanisms working together at the *CDC20* promoter modulated by KMT5A to ensure the timely expression of this gene.

Both KMT5A and CDC20 are essential cell cycle regulator proteins. KMT5A is regulated by ubiquitin-mediated protein turnover specifically at the G1/S transition and between metaphase and anaphase [7] whilst CDC20 regulates the SAC to control the progression from metaphase to anaphase and ensure the successful separation of sister chromatids. It is thought that the methylation of H4K20 is key to successful mitosis with the turnover of KMT5A being the major mode of H4K20 methylation regulation. Indeed, H4K20Me-mediated chromosome condensation is important in this process and *KMT5A* knock out studies resulted in chromosome decondensation leading to cell cycle arrest at G2/M [46]. Furthermore, H4K20Me1 is required for kinetochore assembly at centromeres via the recruitment of CENP-T [53]. Hence, it is logical to hypothesise that a lack of KMT5A, resulting in a decrease in H4K20Me1, will result in impaired kinetochore assembly and thereby will invoke the SAC preventing CDC20 from facilitating the onset of the anaphase. Therefore, due to the importance of tightly regulating *CDC20* to ensure effective mitosis, the modulation of *CDC20* levels themselves by KMT5A would provide a failsafe way to prevent a mitotic catastrophe.

CDC20 is required for nuclear movement prior to the anaphase where its activity, as part of the APC^CDC20^ complex, results in the destruction of cyclin B and the inactivation of CDK1. Interestingly, the CDK1-mediated phosphorylation of KMT5A at serine 29 has been reported to occur during metaphase resulting in the removal of KMT5A from chromatin, holding it in a stabilised state without affecting methylase activity. It is not until anaphase that dephosphorylation by cdc14a/b permits KMT5A protein turnover via APC^cdh1^ [7]. Furthermore, APC^CDC20^ targets the H4K20Me1 demethylase, PHF8, for ubiquitin-mediated destruction [13] further highlighting the important relationship between CDC20 and the enzymes which modulate the H4K20 methylation state.

*CDC20* has been found to be overexpressed in a number of cancers, including prostate cancer [15,54], and there are a number of studies which demonstrate the relevance of *CDC20* to prostate cancer development and progression. For example, *CDC20* has been identified as a hub gene, alongside *CDK1*, in castration-resistant prostate cancer [55], and contributes to cell migration, disease progression and a poorer prognosis in metastatic prostate cancer [56] with another study showing that CDC20, alongside PLK1 and cyclin A, plays a critical role in prostate cancer metastasis [57]. *CDC20* and *PLK1* are both located at chromosomal region 9p, which is often amplified in cancer. Indeed, high expressions of *CDC20*, *PLK1* and *CDK1* correlate with prostate cancer occurrence [58] and worse biochemical recurrence survival rates [59]. Furthermore, CDC20 is a target protein of the Speckle-type POZ protein (SPOP), which functions to promote ubiquitin-mediated protein turnover. *SPOP* is mutated in up to 15% of prostate cancers [60] and these mutations have been shown to result in an inability of SPOP to associate with CDC20, preventing CDC20 protein turnover and consequently resistance to CDC20 inhibitors [16]. Both SPOP mutation [61] and *CDC20* overexpression are important in docetaxel resistance with the inhibition or knockdown of *CDC20* being able to resensitise cells to docetaxel [62] highlighting the importance of CDC20 as a therapeutic target in prostate cancer at several disease stages. With inhibitors for both CDC20 and KMT5A being developed, it would be important to determine whether or not they are able to synergise with each other in drug-resistant models of prostate cancer.

KMT5A has an important role in the DNA damage repair pathway where it is recruited to double-strand breaks to deposit H4K20Me1 to facilitate Suv4-20-mediated H4K20Me2, which is required for 53BP1 binding and successful repair by NHEJ [63,64]. In addition, the ubiquitination of KMT5A by RNF8 increases KMT5A association with RNF168 which in turn promotes H2A ubiquitination [65]. The ubiquitination of these and other chromatin components results in the recruitment of BRCA1/BARD1/Abraxas and RAP80 to sites of γH2AX to allow the repair process to take place. Interestingly, RAP80 is a target of CDC20 and its overexpression prevents mitotic progression irrespective of DNA damage [9]. This again supports a connection between the functions of KMT5A and CDC20 in cellular processes. Additionally, the role of KMT5A in the suppression of important anti-tumourigenic processes such as the positive regulation of the apoptotic process and the response to gamma and ionising irradiation are also highlighted suggesting the utility of KMT5A inhibition in combination with other DNA damage-inducing therapeutics such as radiotherapy or cytotoxic agents.

The cellular processes regulated by KMT5A identified in this study are consistent with those already described such as genome integrity, cell cycle progression, gene transcription and DNA damage repair. However, some novel processes were identified including RNA splicing and mRNA processing which require further investigation. This is particularly important in prostate cancer where aberrant RNA splicing, particularly of the androgen receptor, is associated with therapy resistance and poor prognosis [66].

## 5. Conclusions

A number of key oncogenic signalling pathways are regulated by the methyltransferase KMT5A. Here, we provide evidence of the role of KMT5A in both metaphase to anaphase control via the regulation of CDC20 and propose that close links between mitosis and DNA damage repair processes are present via this relationship. As both *CDC20* and *KMT5A* are up-regulated in cancer via a number of mechanisms, the relationship between the two proteins may be dysregulated, thereby promoting genomic instability. This presents an opportunity to identify beneficial therapeutic combinations to treat patients based on a number of criteria such as *SPOP* mutation status, *KMT5A* expression status and therapeutic resistance.

## Data Availability

The microarray data presented in this study are openly available in GEO: GSE233350. Publicly available datasets were analysed in this project. This data can be found in cBioportal.

## Supplementary Materials

The following supporting information can be downloaded at https://www.mdpi.com/article/10.3390/cancers15143597/s1. Supplementary Figure S1. KMT5A and CDC20 knockdown inhibits proliferation of prostate cancer cells. Supplementary Figure S2. KMT5A inhibition by UNC0379 reduces KMT5A levels and H4K20Me1. Supplementary Figure S3. KMT5A inhibition by Ryuvidine reduces KMT5A levels and H4K20Me1. Supplementary Figure S4. KMT5A knockdown causes DNA damage. Supplementary Figure S5. KMT5A levels remain constant in response to DHT stimulation. Supplementary Figure S6. Acetylation and phosphorylation of p53 occurs upon KMT5A knockdown. Table S1. qPCR primers. Table S2. Genes significantly up-regulated by KMT5A knockdown. Table S3. Genes significantly down-regulated by KMT5A knockdown. Table S4. KEGG pathways down-regulated in response to KMT5A knockdown in the presence of DHT stimulation. Table S5. KEGG pathways up-regulated in response to KMT5A knockdown in the presence of DHT stimulation. Table S6. Biological processes down-regulated in response to KMT5A knockdown in the presence of DHT stimulation. Table S7. Biological processes up-regulated in response to KMT5A knockdown in the presence of DHT stimulation. File S1. Uncut blots.
