# Supplementary material for: CDC20 Is Regulated by the Histone Methyltransferase, KMT5A, in Castration-Resistant Prostate Cancer
Source: Cancers (Basel). 2023 Jul 13;15(14):3597. doi: 10.3390/cancers15143597 (PMC10377584; doi:10.3390/cancers15143597)
Supplement: Supplementary file 1 [file cancers-15-03597-s001.zip › Supplementary Figures.pdf]

Supplementary Figures

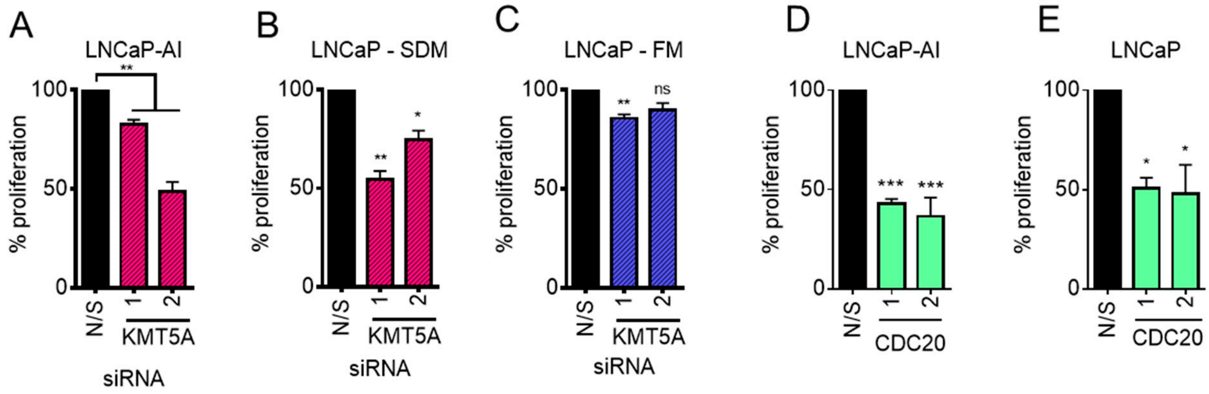

Supplementary Figure S1. KMT5A and CDC20 knockdown inhibits proliferation of prostate cancer cells. (A) LNCaP-AI and (B) LNCaP cells were reverse transfected with 2 independent siRNAs targeting KMT5A in steroid depleted media (SDM) and (C) LNCaP cells in full media then allowed to proliferate for 3 doubling times prior to assessment of proliferation by SRB assay. (D) LNCaP-AI and (E) LNCaP cells were reverse transfected with 2 independent siRNAs targeting CDC20 in their respective growth media. Cells were allowed to proliferate for 3 doubling times prior to assessment of proliferation by SRB assay. One-way ANOVA defined statistical significance * $p<0.05$; ** $p<0.01$;*** $p<0.001$

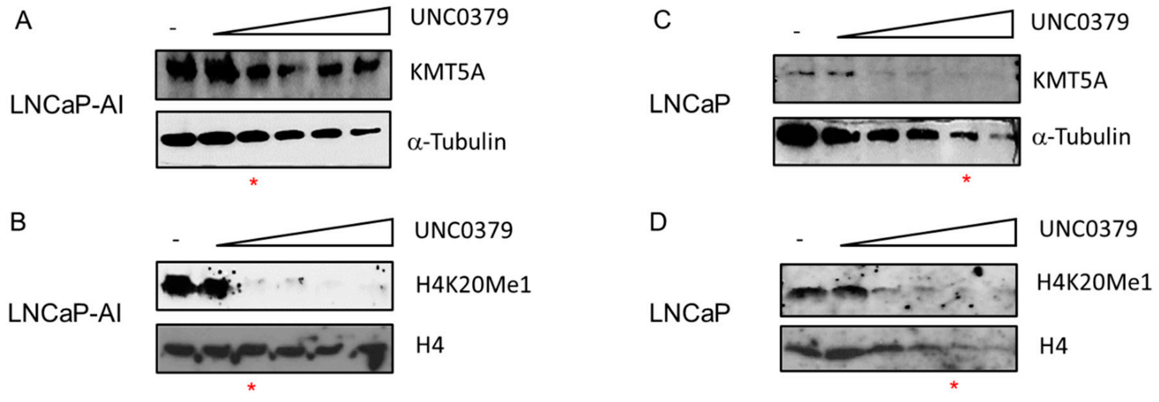

Supplementary Figure S2. KMT5A inhibition by UNC0379 reduces KMT5A levels and H4K20Me1. LNCaP-AI were treated with 0, 2, 4, 6, 7 and 8 μM UNC0379 for 48 hours then

(A) KMT5A and (B) H4K20Me1 were assessed by western blotting. (C) LNCaP cells were treated with the same doses for 48 hours prior to western blotting for KMT5A and (D) H4K20Me1. *denotes ~GI50 concentration.

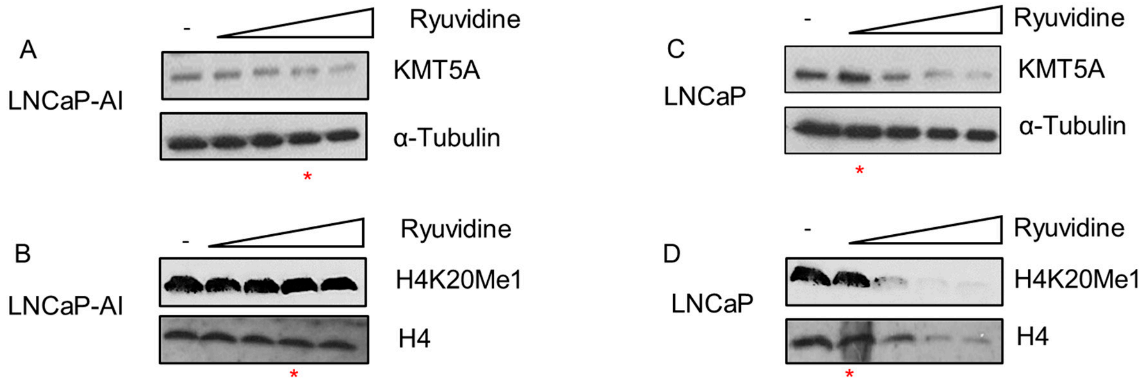

Supplementary Figure S3. KMT5A inhibition by Ryuvidine reduces KMT5A levels and H4K20Me1. LNCaP-AI were treated with 0, 1, 1.5, 2, 2.5 and 3 μM Ryuvidine for 48 hours then (A) KMT5A and (B) H4K20Me1 were assessed by western blotting. (C) LNCaP cells were treated with the same doses for 48 hours prior to western blotting for KMT5A and (D) H4K20Me1. *denotes ~GI50 concentration.

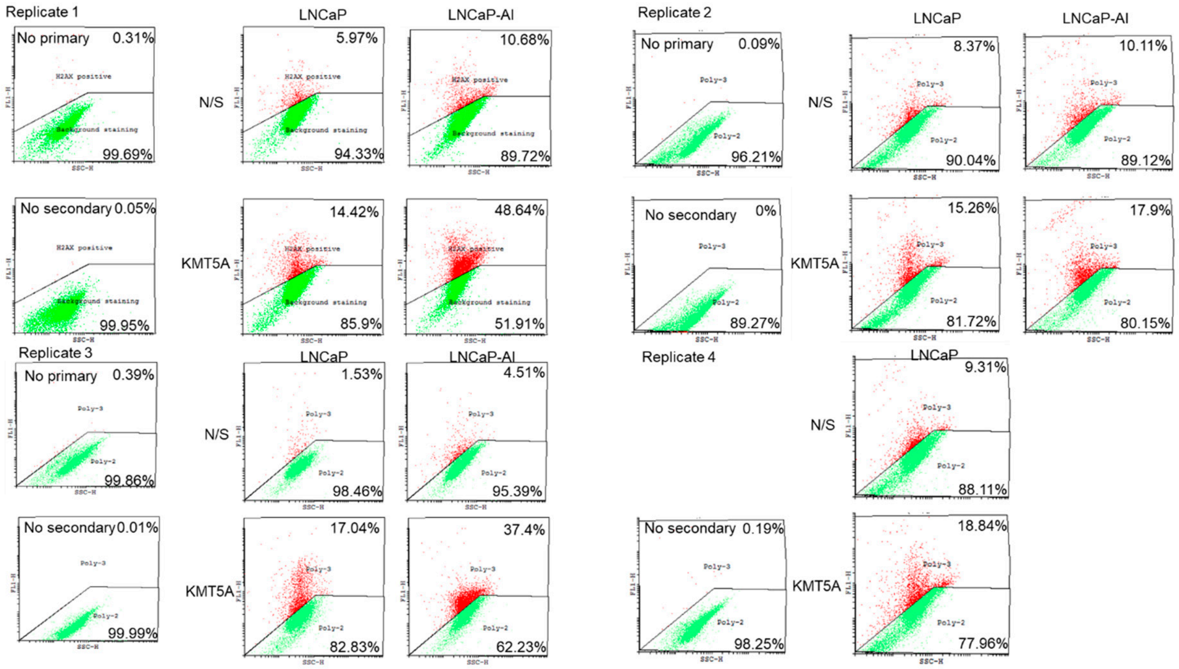

Supplementary Figure S4. KMT5A knockdown causes DNA damage. LNCaP-AI and LNCaP cells were reverse transfected with a pool of 2 KMT5A targeting siRNAs for 72 hours. Cells were collected and stained for γH2AX prior to analysis by flow cytometry. Scatter plots are shown for 4 independent experimental replicates for LNCaP cells and 3 replicates for LNCaP-AI cells.

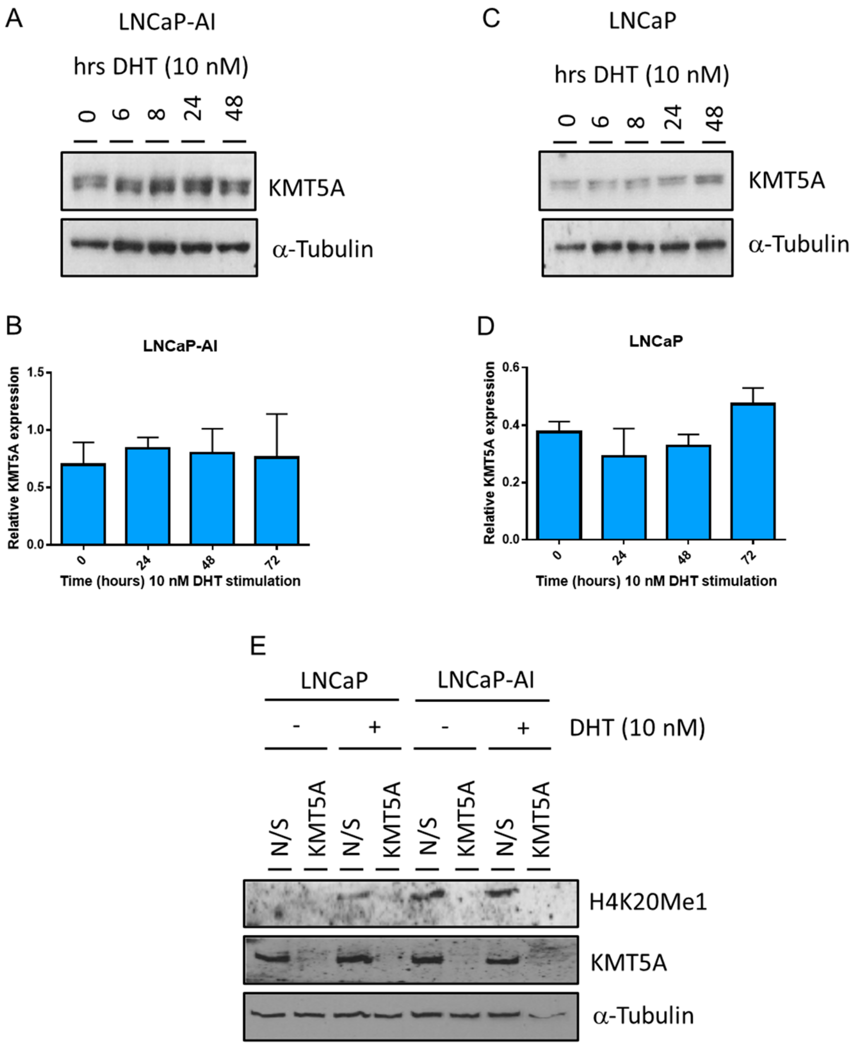

Supplementary Figure S5. KMT5A levels remain constant in response to DHT stimulation. (A) LNCaP-AI cells were seeded out in steroid depleted media and allowed to adhere for 48 hours prior to stimulation with 10 nM DHT for the indicated times. Protein lysates and (B) RNA were then harvested and assessed for KMT5A levels by western blotting and qPCR respectively. (C) LNCaP cells were seeded out under the same experimental treatments as in (A) and (B) with the same downstream experimentation. Western blots show

representative images of at least 4 experimental repeats and qPCR data show relative KMT5A expression normalised to HPRT1 housekeeping gene averaged from 2 experimental repeats for LNCaP-AI and 4 experimental repeats for LNCaP cells. One-way ANOVA statistical test on this data reveals no statistically significant difference in expression with exposure time to DHT. (E) LNCaP and LNCaP-AI cells were reverse transfected with siRNA targeting KMT5A or a non-silencing control (N/S) in steroid depleted media and incubated for 72 hours prior to addition of 10 nM DHT for a further 24 hours. Protein lysates were collected and analysed by western blotting.

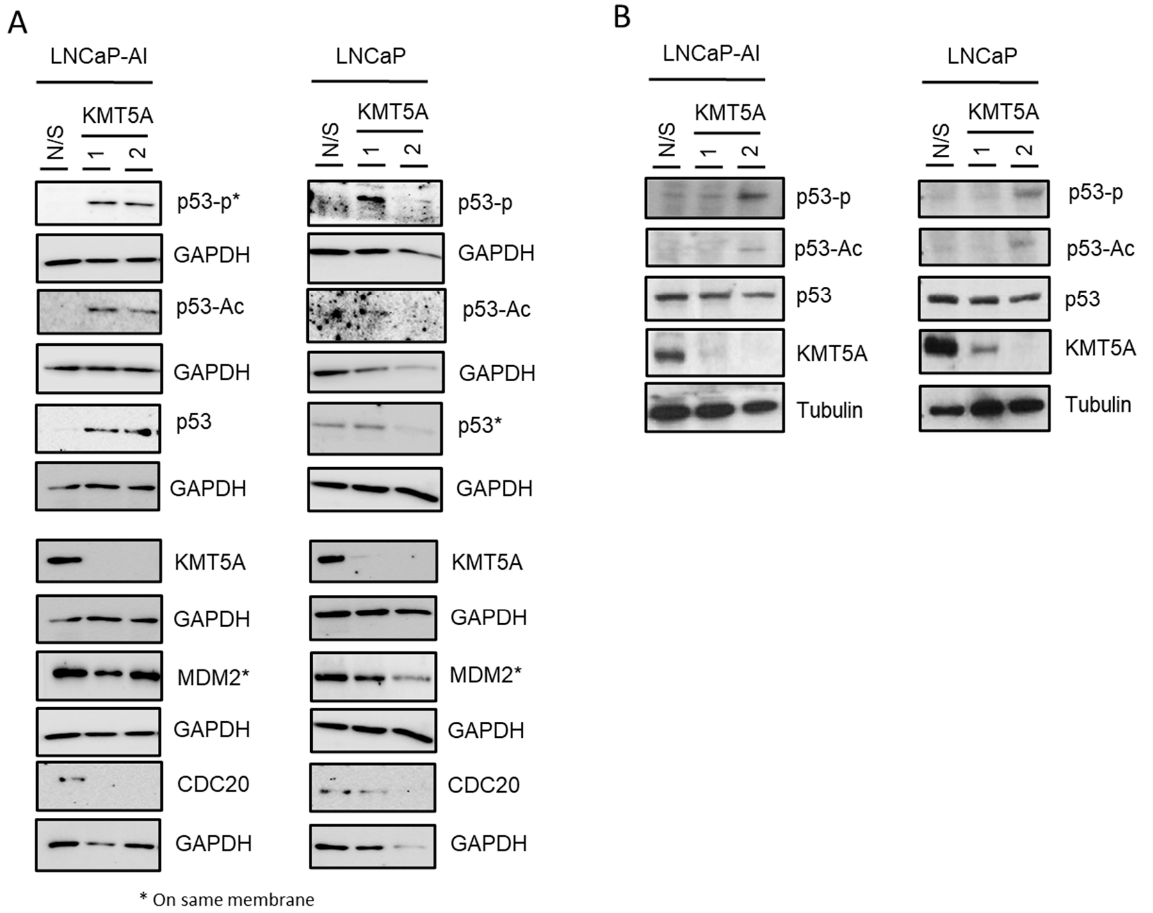

Supplementary Figure S6. Acetylation and phosphorylation of p53 occurs in response to KMT5A knockdown. (A) LNCaP-AI and LNCaP cells were reverse transfected with 2 independent siRNA sequences for 72 hours prior to western blotting analysis for CDC20, p53, p-p53, p53-Ac, MDM2, KMT5A. GAPDH was used as a loading control. ChemiDoc (BioRad)

images are shown. *p53-p, MDM2 and p53, MDM2 were probed on the same membrane for LNCaP-AI and LNCaP samples, respectively and therefore have the same GAPDH loading control. (B) Earlier experimental repeat of the same experiment as shown in (A) except only p-p53, p53-Ac, p53, KMT5A and tubulin were assessed. Results were developed on X-ray film.
