# Supplementary material for: CDC20 Is Regulated by the Histone Methyltransferase, KMT5A, in Castration-Resistant Prostate Cancer
Source: Cancers (Basel). 2023 Jul 13;15(14):3597. doi: 10.3390/cancers15143597 (PMC10377584; doi:10.3390/cancers15143597)
Supplement: Supplementary file 1 [file cancers-15-03597-s001.zip › Supplementary File S1. Uncut blots.pdf]

Uncut blots for Figure 2; Lab book 4 p0037

5 minute exposure

50kDa

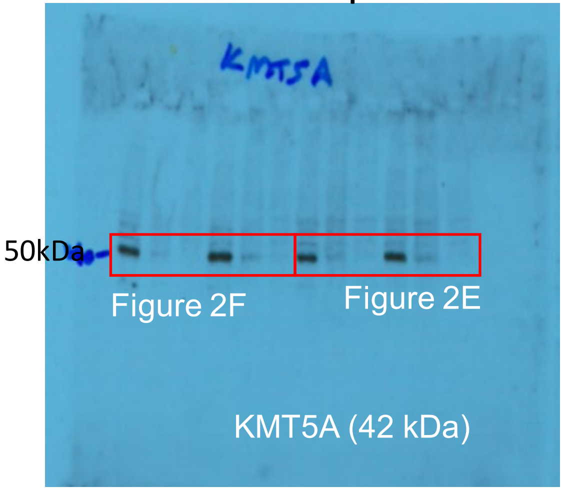

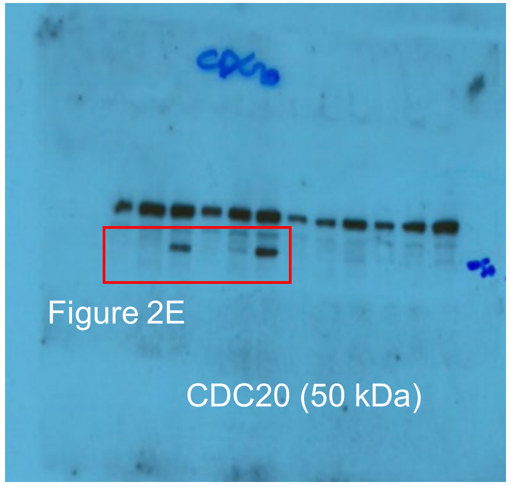

50kDa

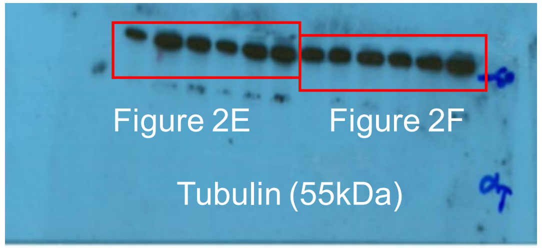

50kDa

10 minute exposure

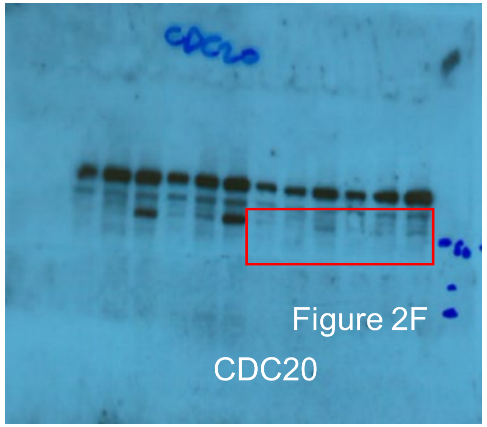

50kDa

1. Marker
2. LNCaP N/S SDM
3. LNCaP KMT5A_1 SDM
4. LNCaP KMT5A_2 SDM
5. LNCaP N/S + 10nM DHT 24h
6. LNCaP KMT5A_1 + 10nM DHT 24h
7. LNCaP KMT5A_2 + 10nM DHT 24h
8. LNCaP-AI N/S SDM
9. LNCaP-AI KMT5A_1 SDM
10. LNCaP-AI KMT5A_2 SDM
11. LNCaP-AI N/S + 10nM DHT 24h
12. LNCaP-AI KMT5A_1 + 10nM DHT 24h
13. LNCaP-AI KMT5A_2 + 10nM DHT 24h

ZA

Uncut blots for Figure 3A; ChemiDoc (BioRad) images

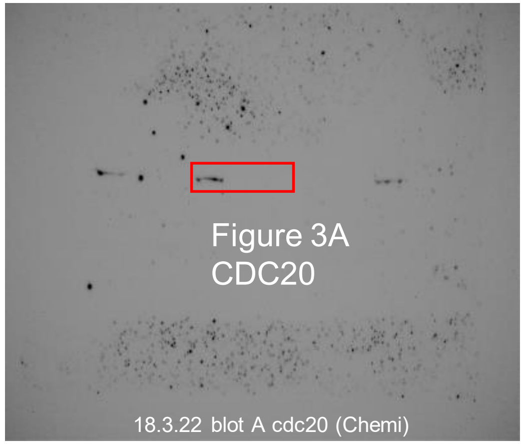

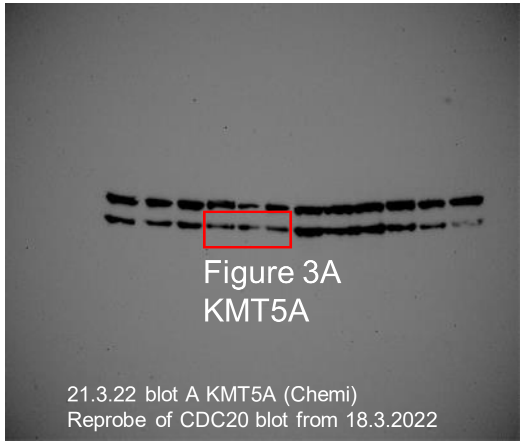

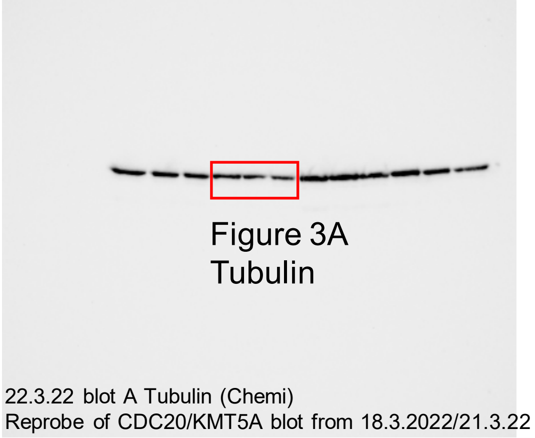

1. Marker
2. LNCaP N/S
3. LNCaP KMT5A_1
4. LNCaP KMT5A_2
5. LNCaP-AI N/S SDM
6. LNCaP-AI KMT5A_1 SDM
7. LNCaP-AI KMT5A_2 SDM
8. LNCaP N/S
9. LNCaP KMT5A_1
10. LNCaP KMT5A_2
11. LNCaP-AI N/S SDM
12. LNCaP-AI KMT5A_1 SDM
13. LNCaP-AI KMT5A_2 SDM

N=3.3.22 (lanes 2–7)

N =7.3.22 (lanes 8–13)

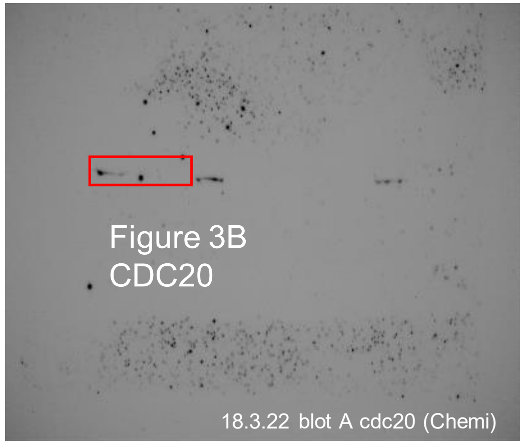

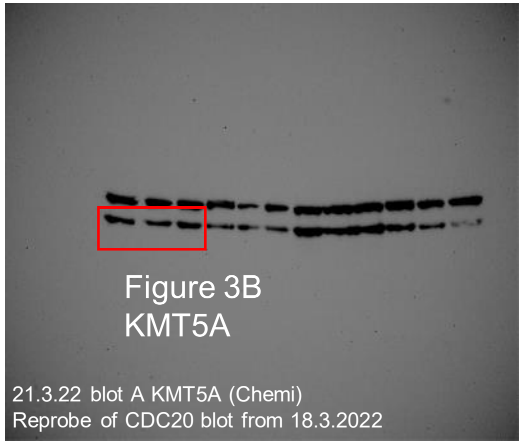

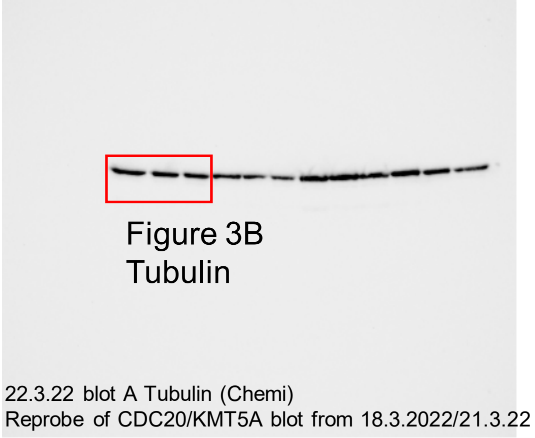

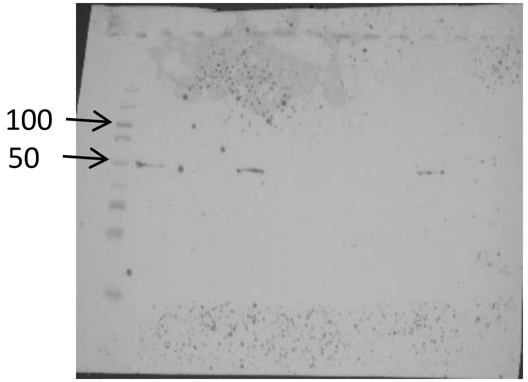

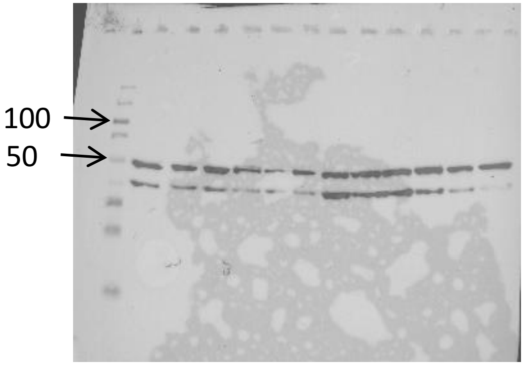

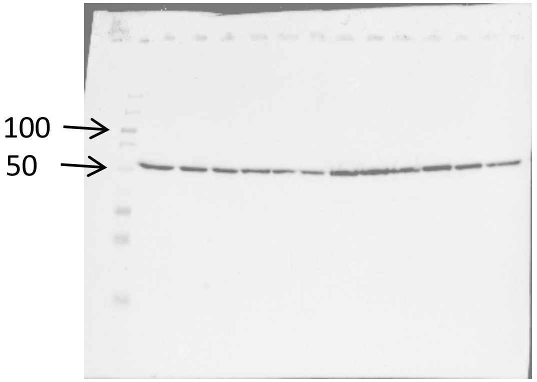

Composite images showing molecular weight marker

Thermofisher Spectra Multi colour Broad Range Protein Marker

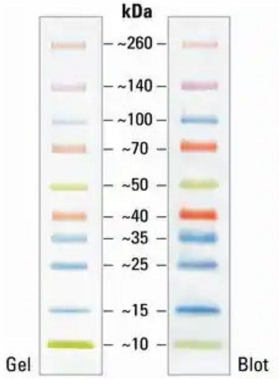

KC

Uncut blots for Figure 5B; lab book 3 p0074

1. Marker
2. LNCaP DMSO
3. LNCaP 7 μM UNC0379
4. LNCaP-AI DMSO
5. LNCaP-AI 4 μM UNC0379

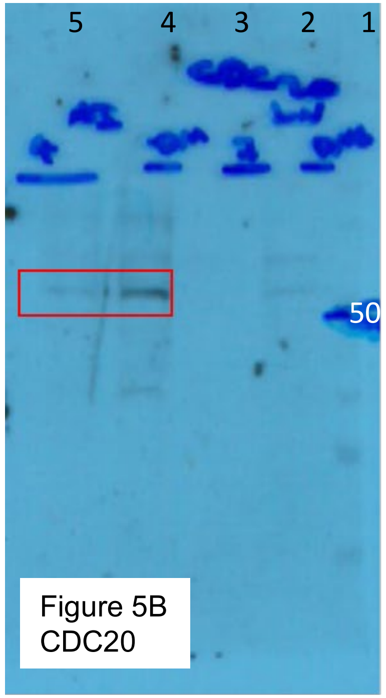

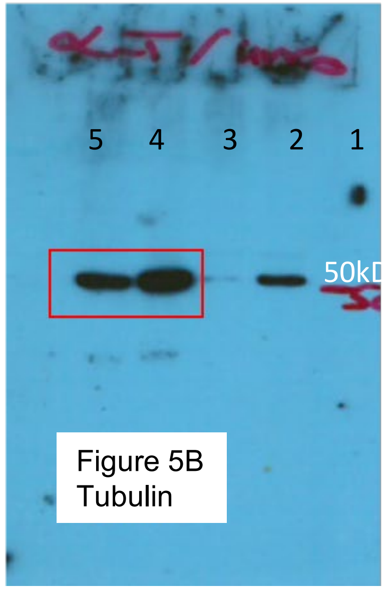

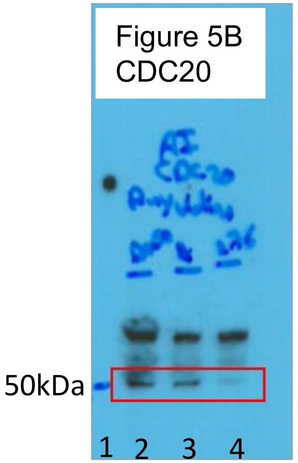

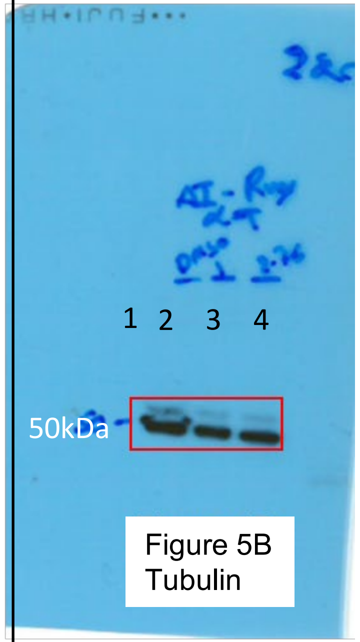

1. Marker
2. LNCaP-AI DMSO
3. LNCaP-AI 1 μM Ryuvidine
4. LNCaP-AI 2.77 μM Ryuvidine

ZA

Uncut blots for Figure 5C; lab book 3 p0074

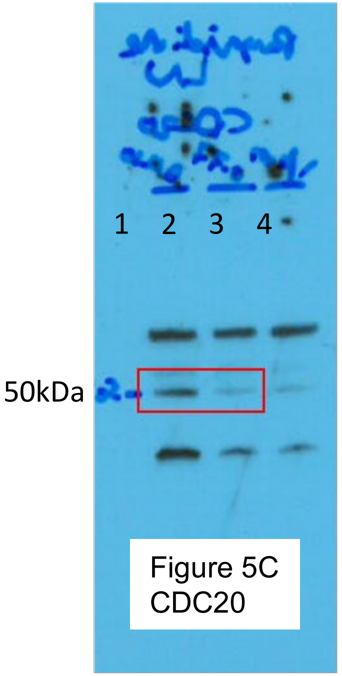

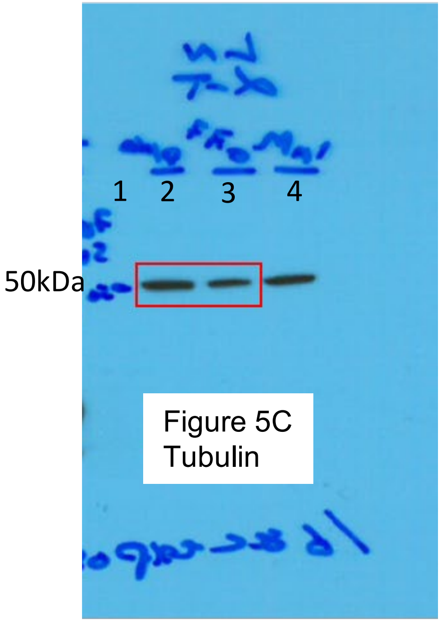

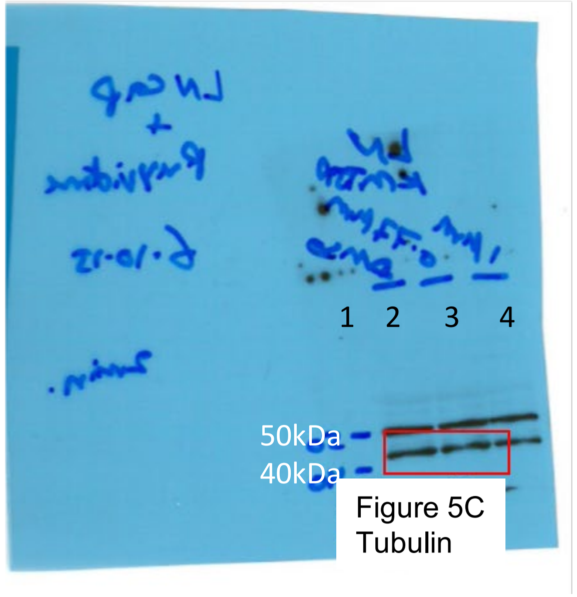

1. Marker
2. LNCaP DMSO
3. LNCaP 0.77 µM Ryuvidine
4. LNCaP 1 µM Ryuvidine

ZA

Uncut blots for Figure 7B; LNCaP-AI

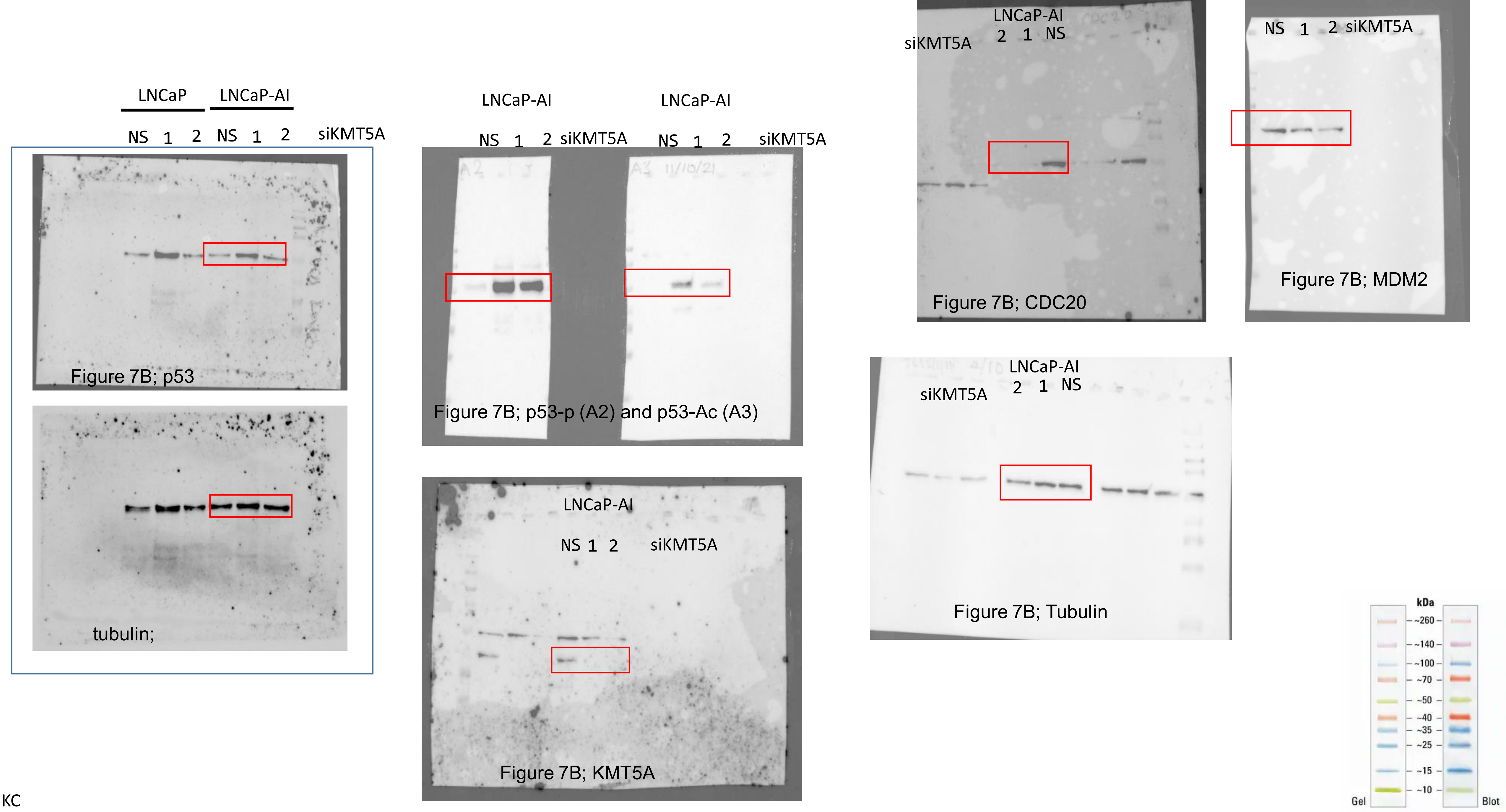

KC

Uncut blots for Figure 7B; LNCaP

LNCaP LNCaP-AI

NS 1 2 NS 1 2 siKMT5A

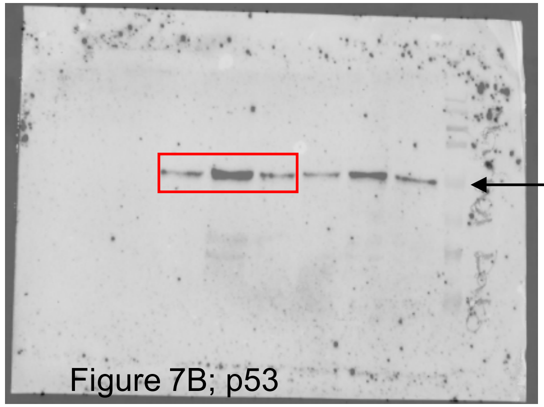

← 50 kDa

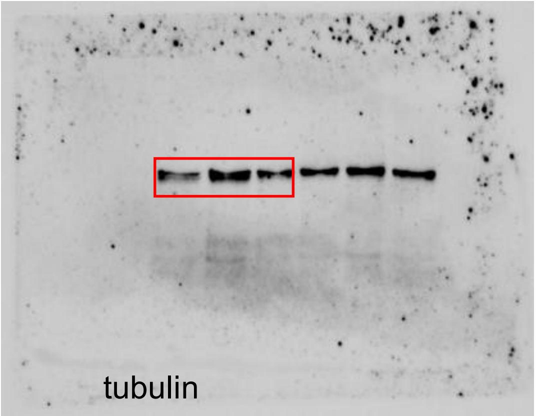

LNCaP

2 1 NS 2 1 NS siKMT5A

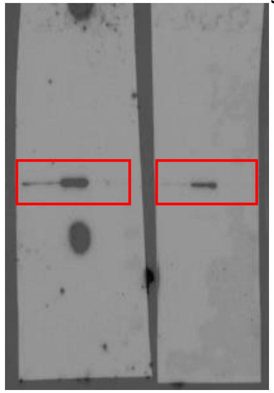

Figure 7B; p53-p (A2) and p53-Ac (A3)

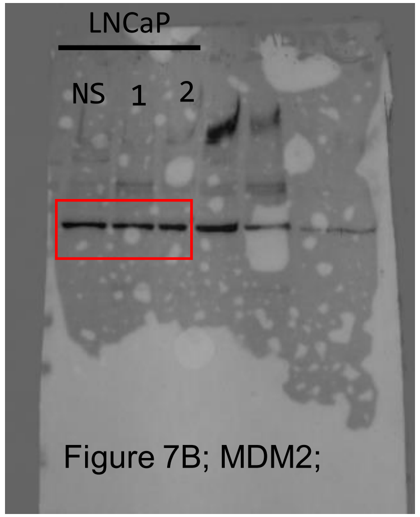

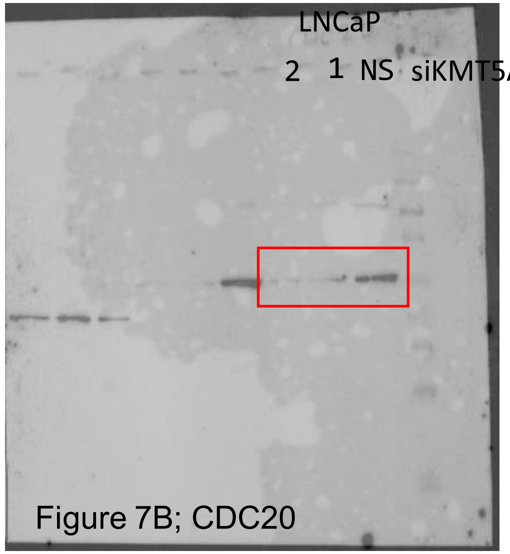

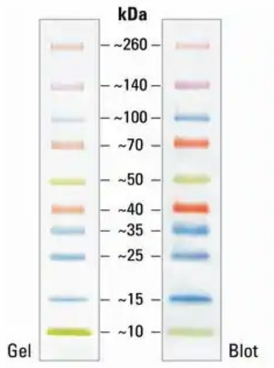

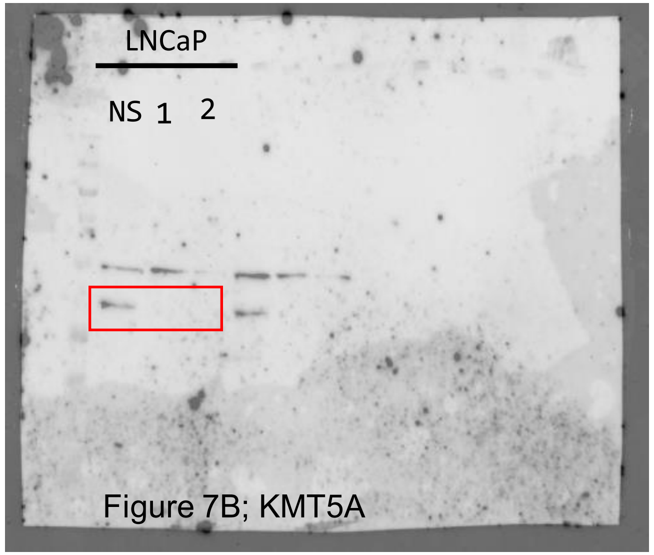

LNCaP

2 1 NS siKM

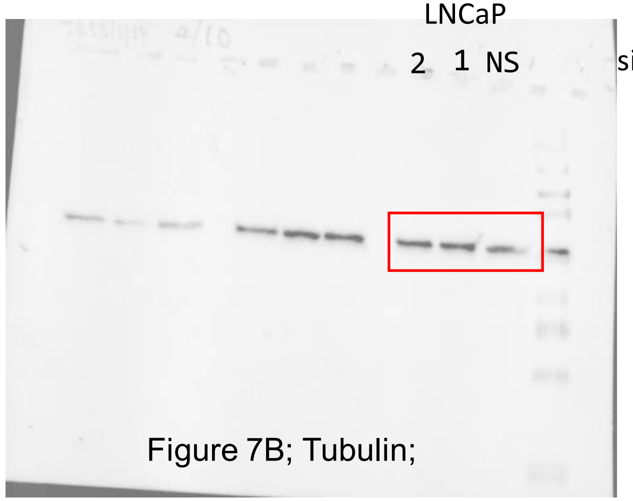

KC, KJ, HC

Uncropped blots for Figure 7D;

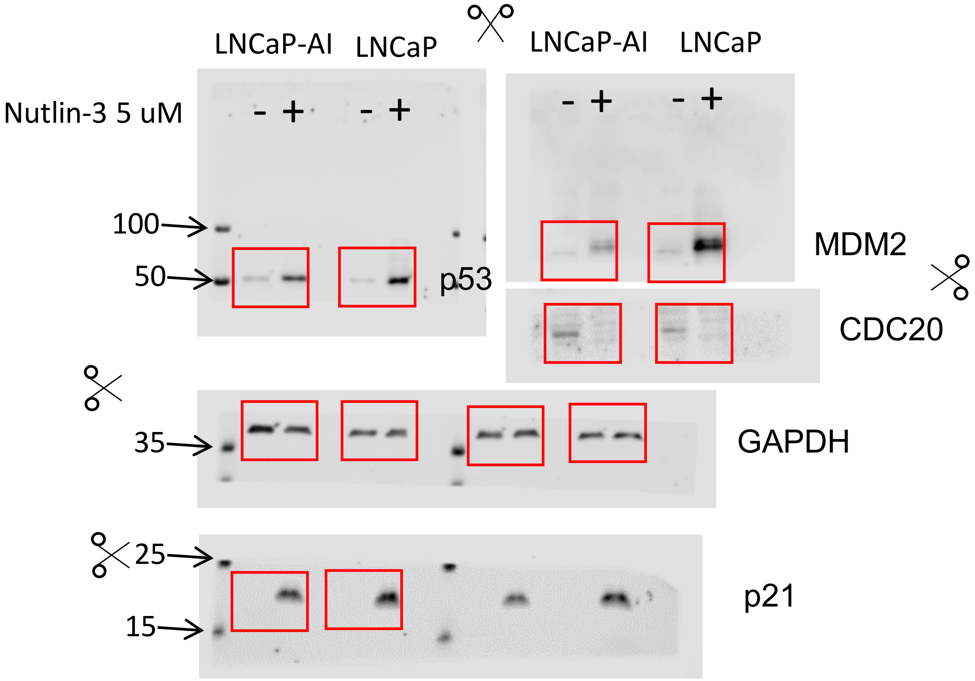

RG; KC

Uncropped blots for Figure 8

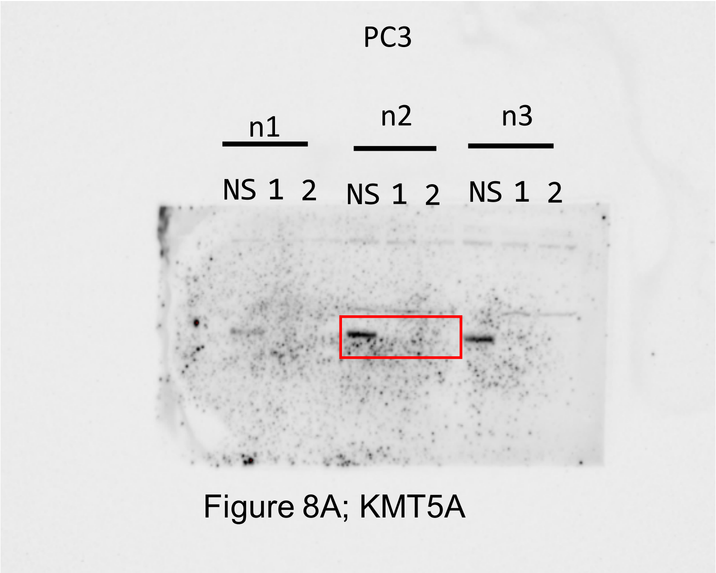

Figure 8A; KMT5A

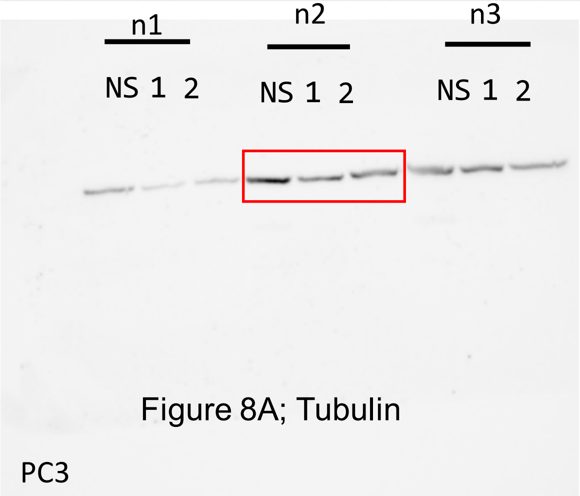

Figure 8A; Tubulin

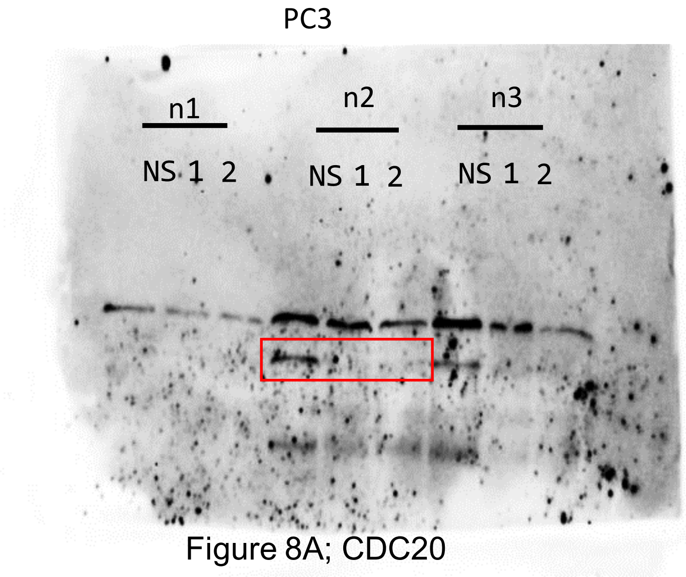

Figure 8A; CDC20

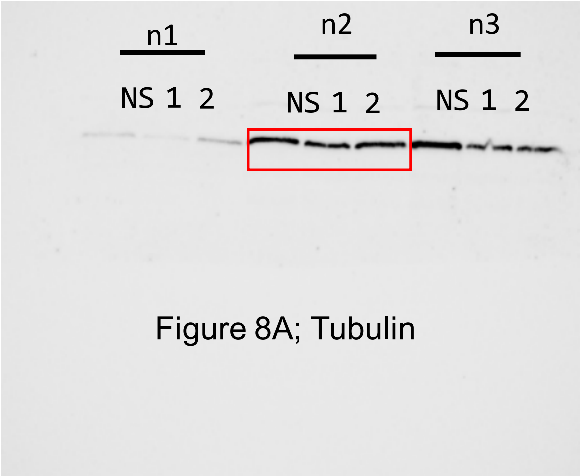

Figure 8A; Tubulin

DA

Supplementary Figure 2 uncut blots

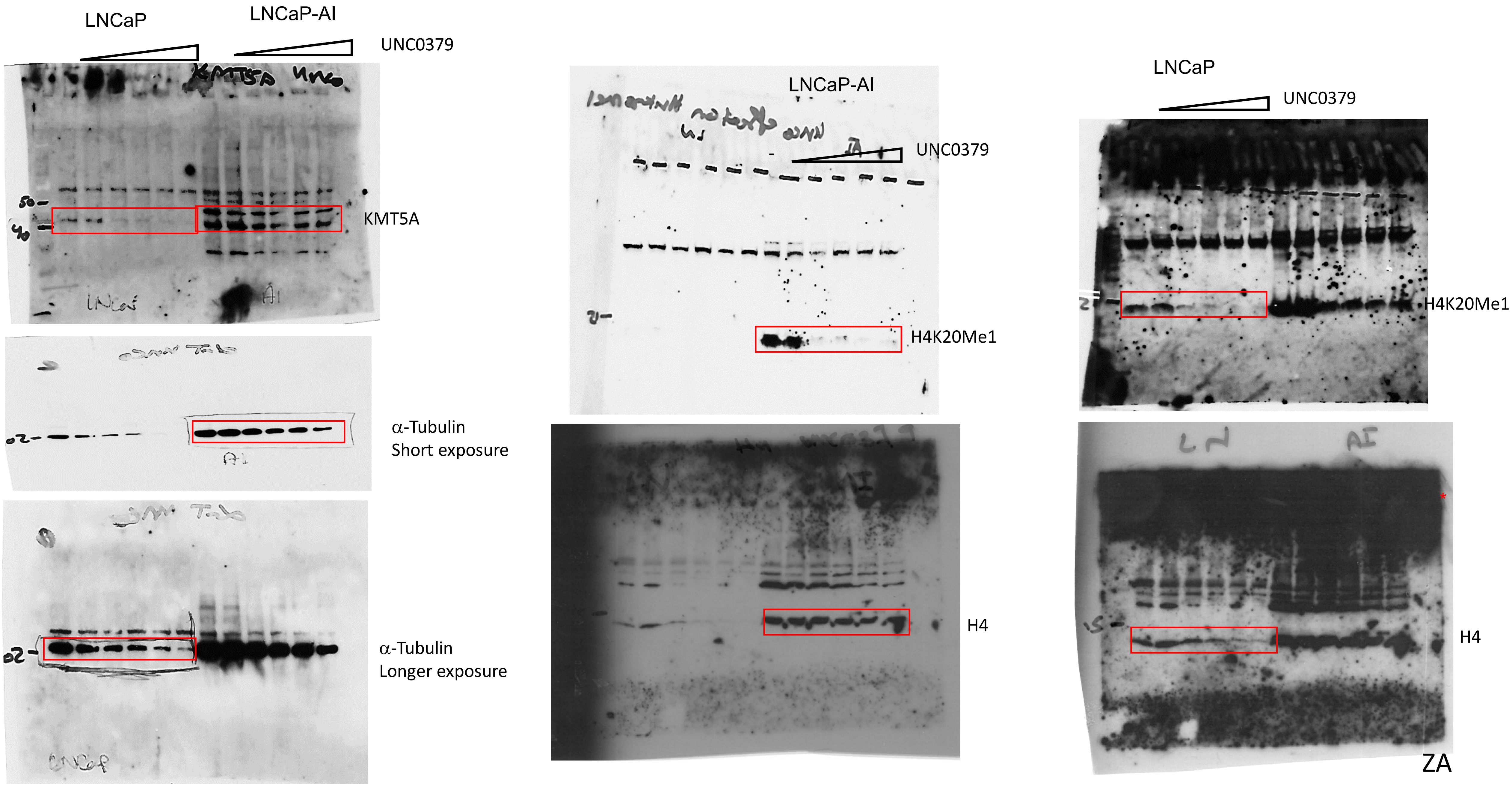

Supplementary Figure 3 uncut blots

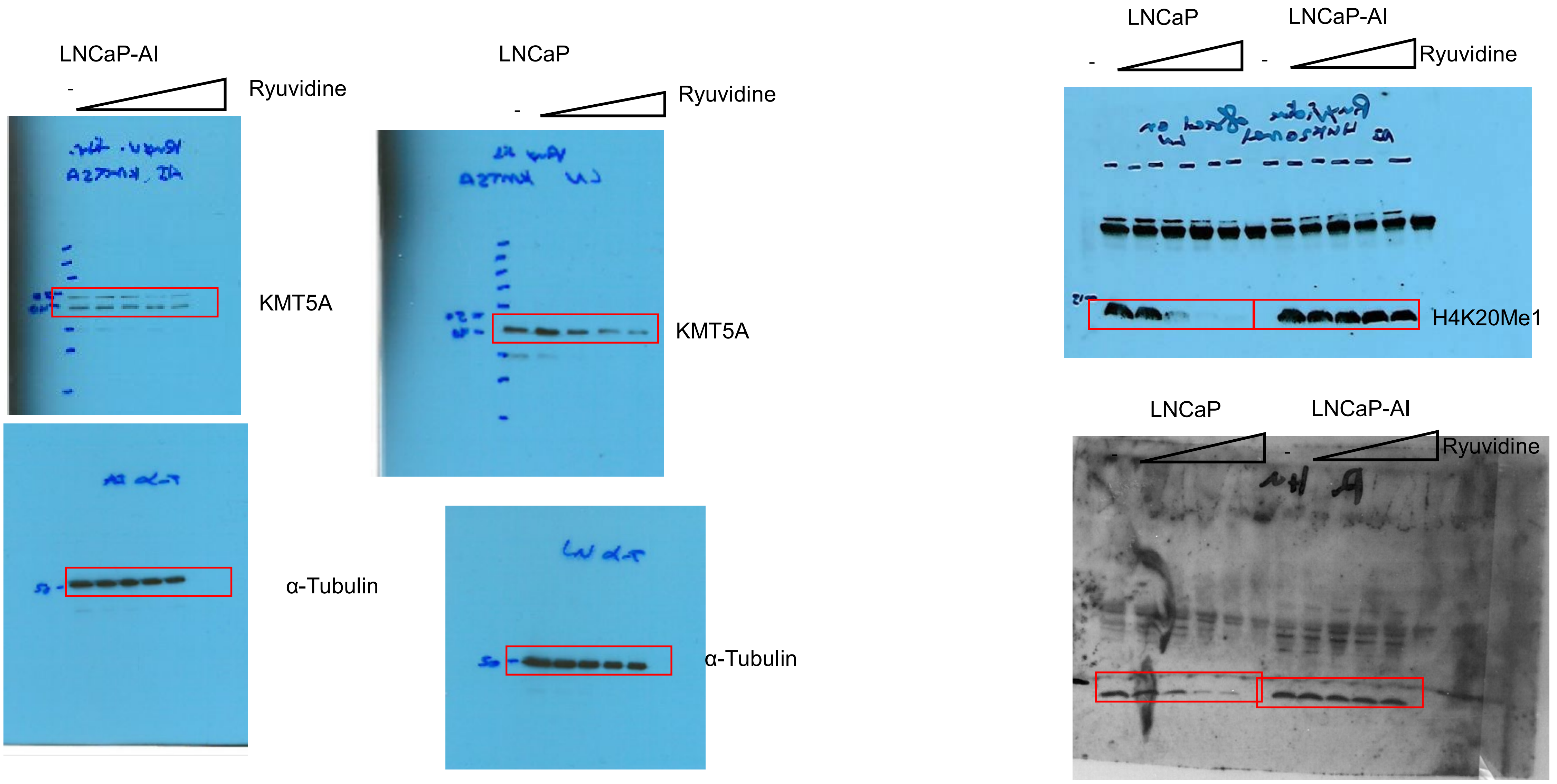

ZA

Supplementary Figure 5 uncut blots

LNCaP

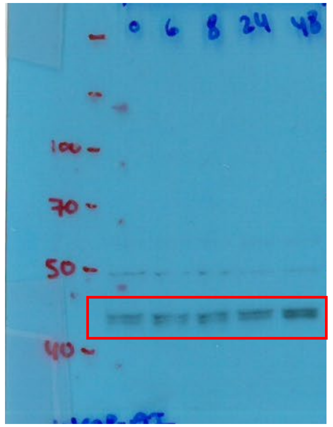

LNCaP

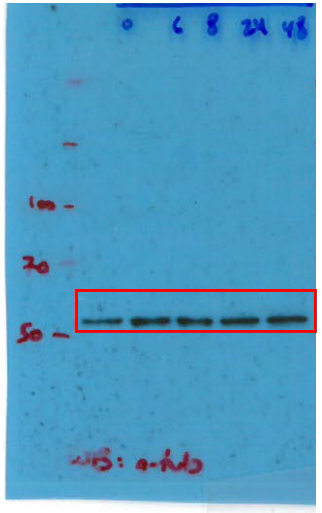

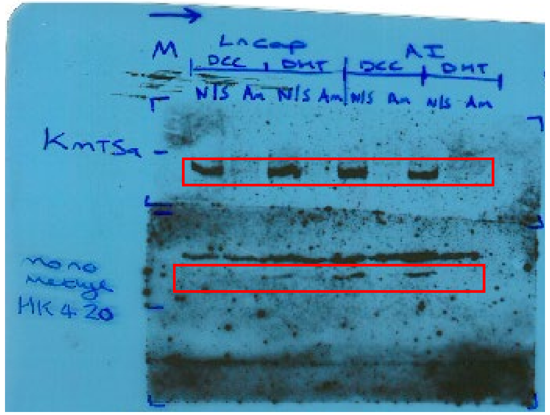

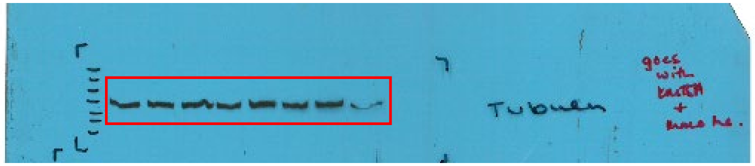

LNCaP-AI

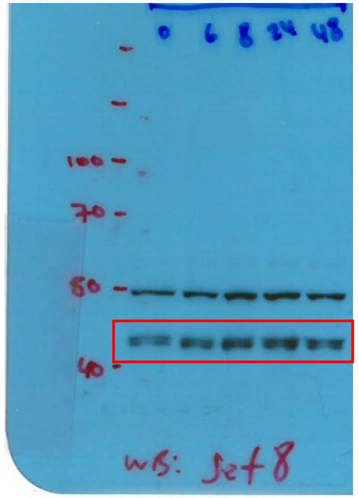

LNCaP-AI

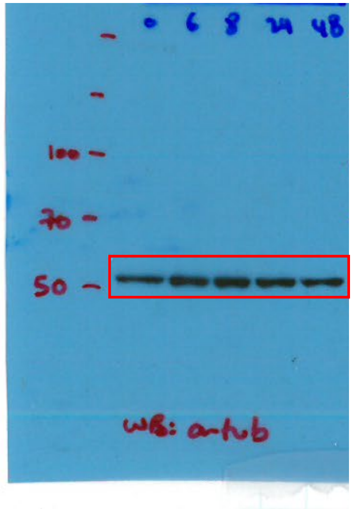

MA, SW

Supplementary Figure 6A uncut blots

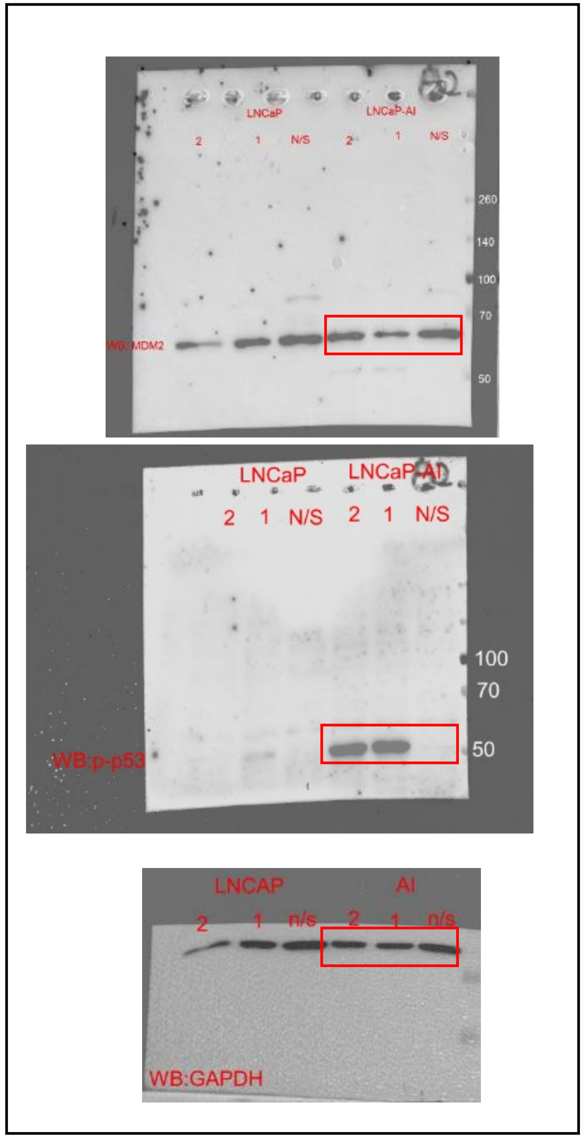

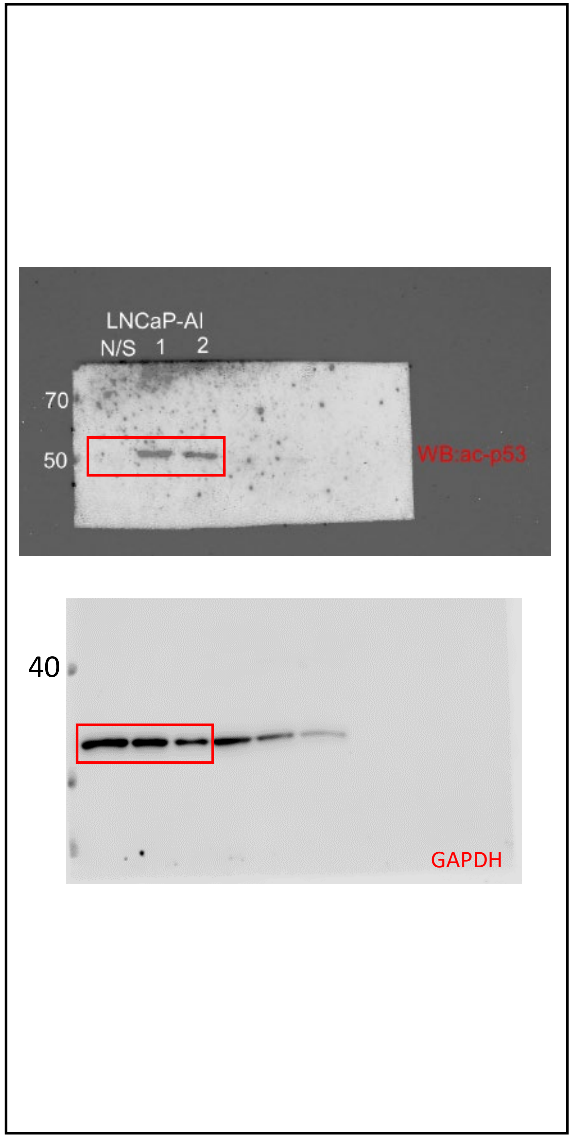

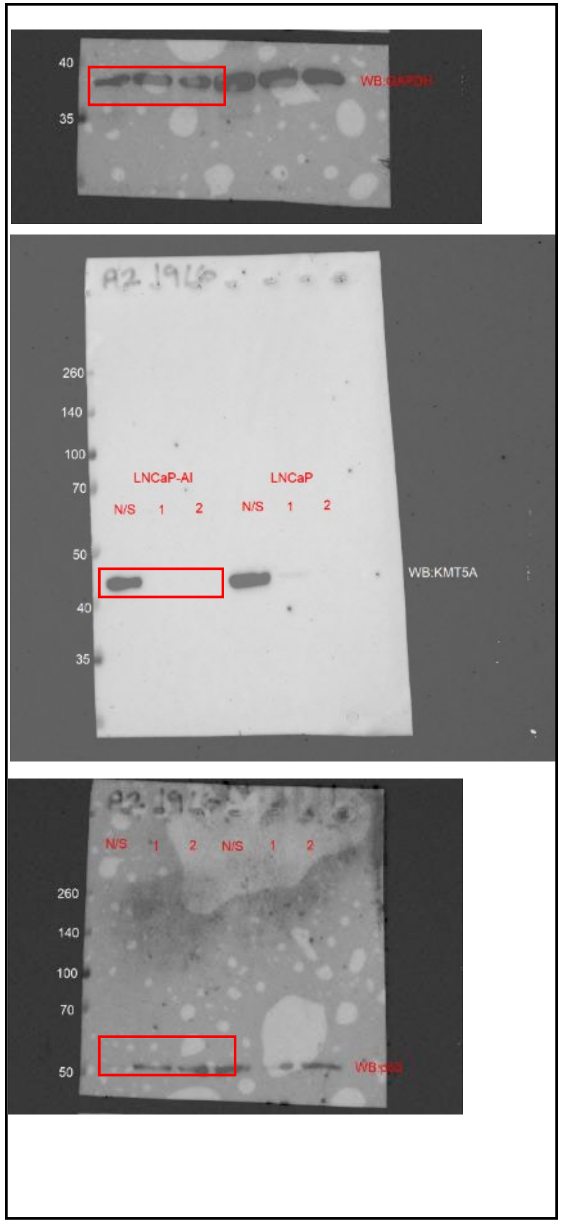

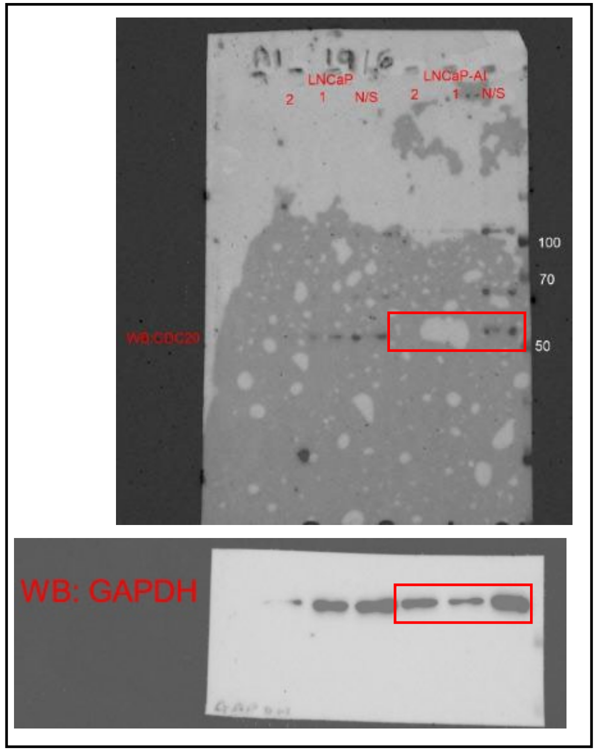

LNCaP-AI uncut blot experimental repeat 3

KC, EL

Supplementary Figure 6A uncut blots

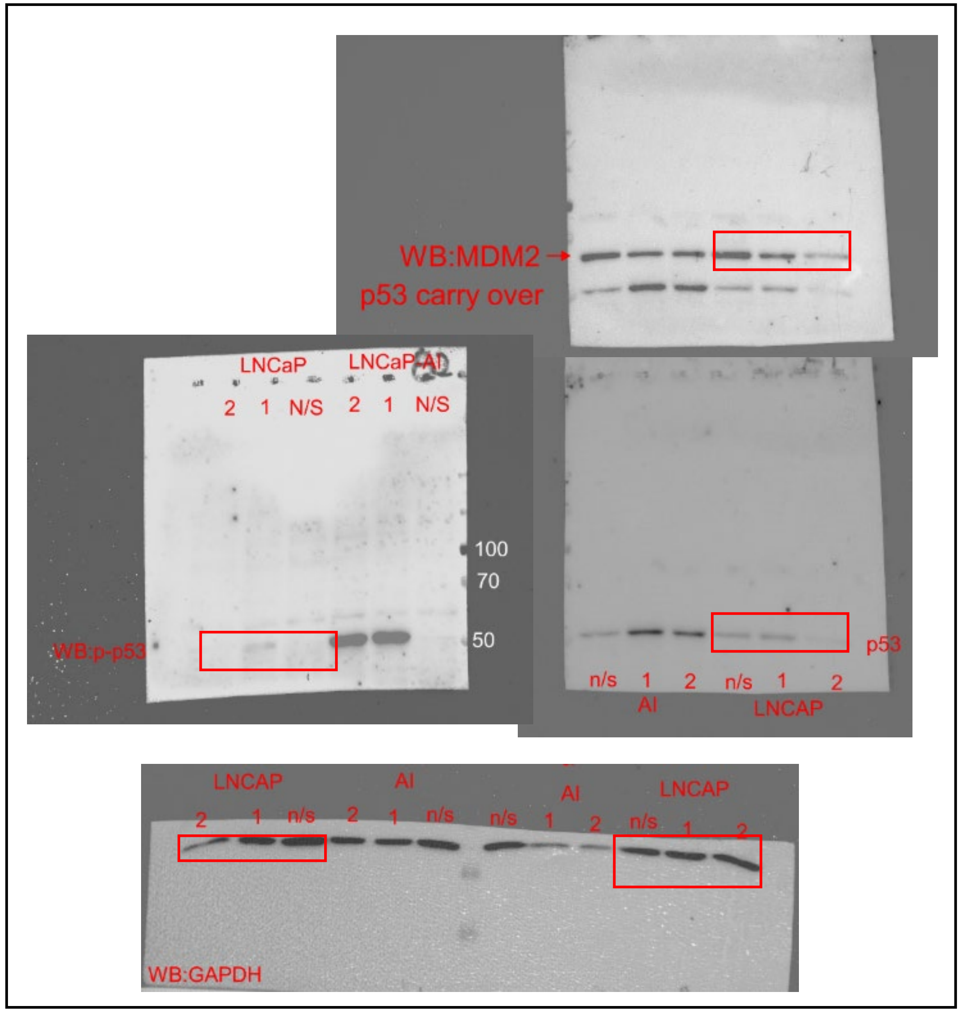

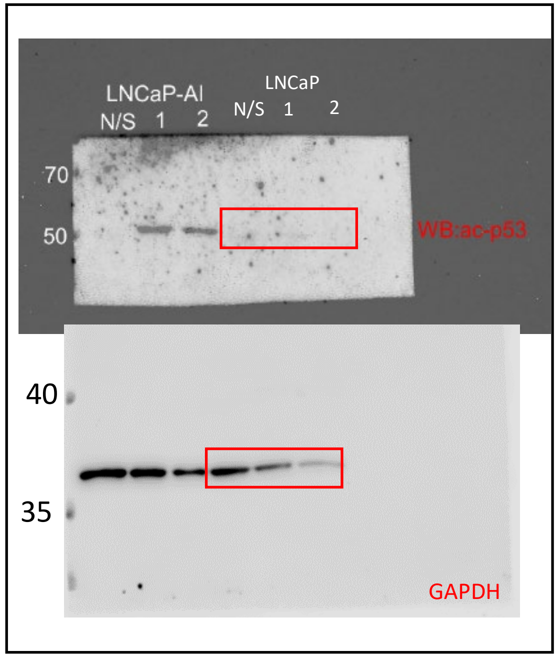

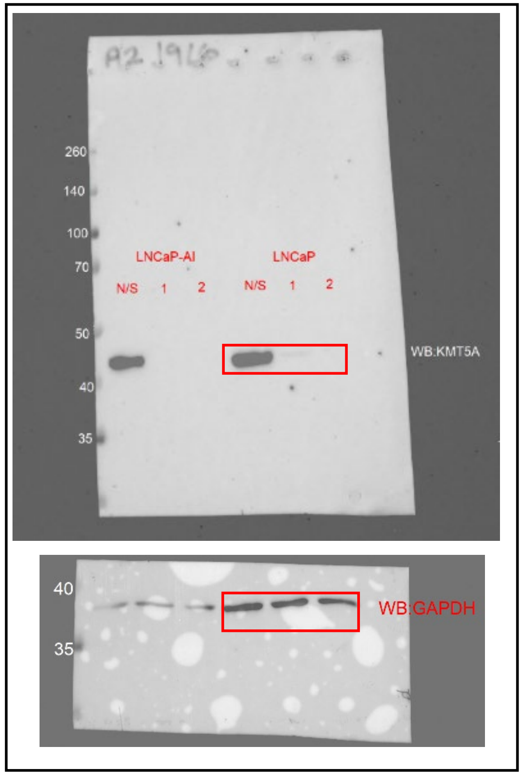

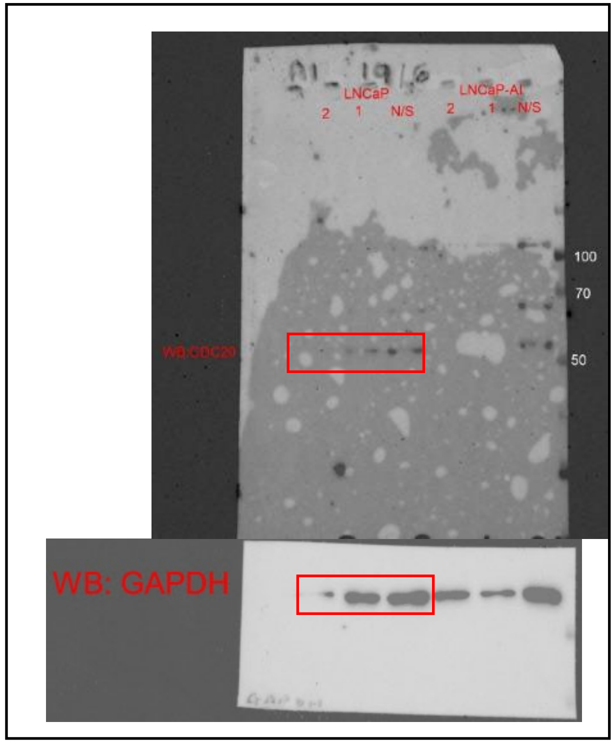

LNCaP uncut blot experimental repeat 3

KC, EL

Supplementary Figure 6B uncut blots

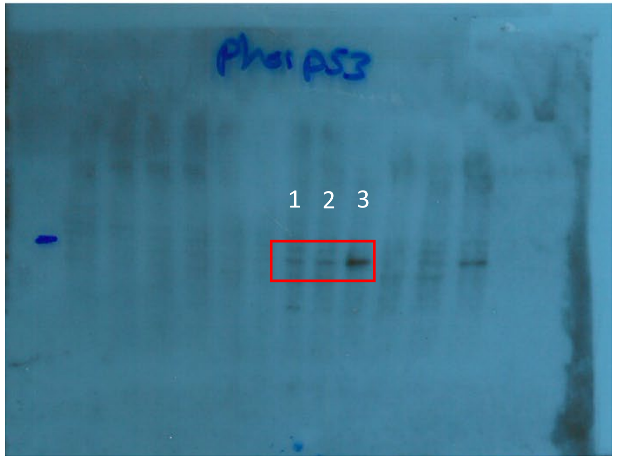

P53-p

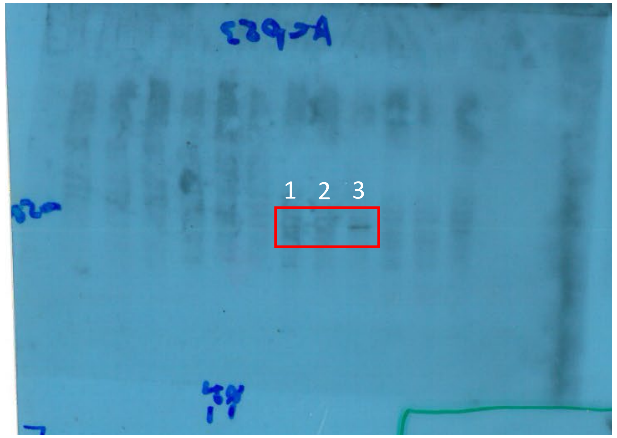

P53-Ac

50

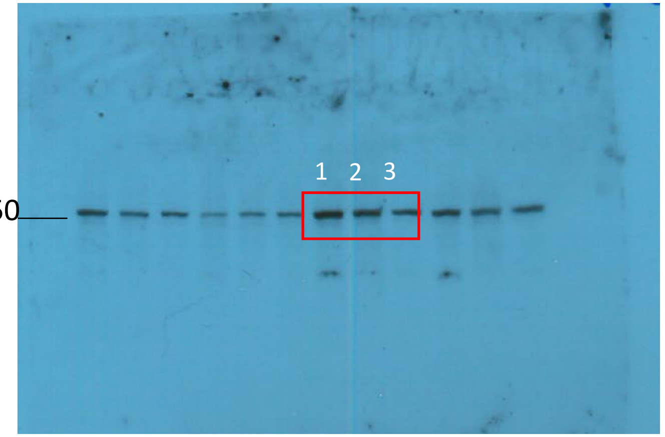

p53

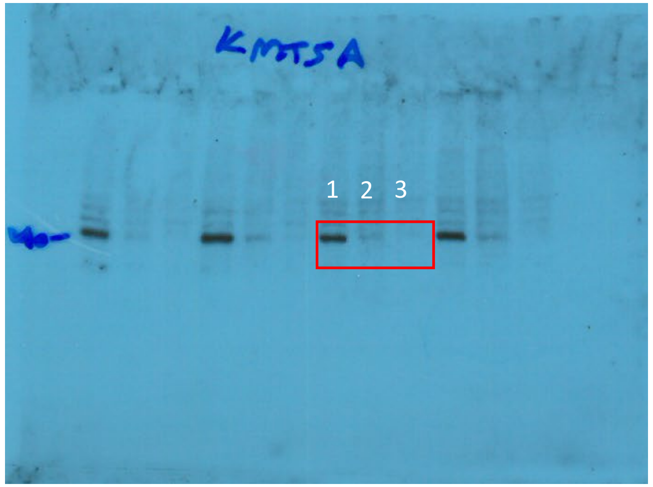

KMT5A

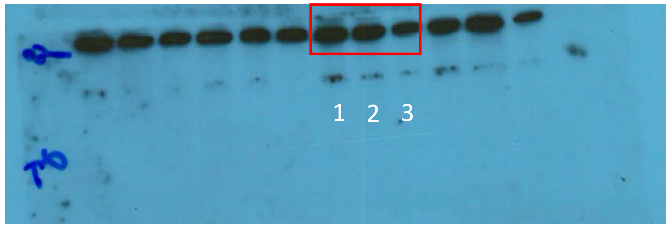

Tubulin

1. N/S
2. siKMT5A_1
3. siKMT5A_2

LNCaP-AI uncut blot experimental repeat 1

ZA

Supplementary Figure 6B uncut blots

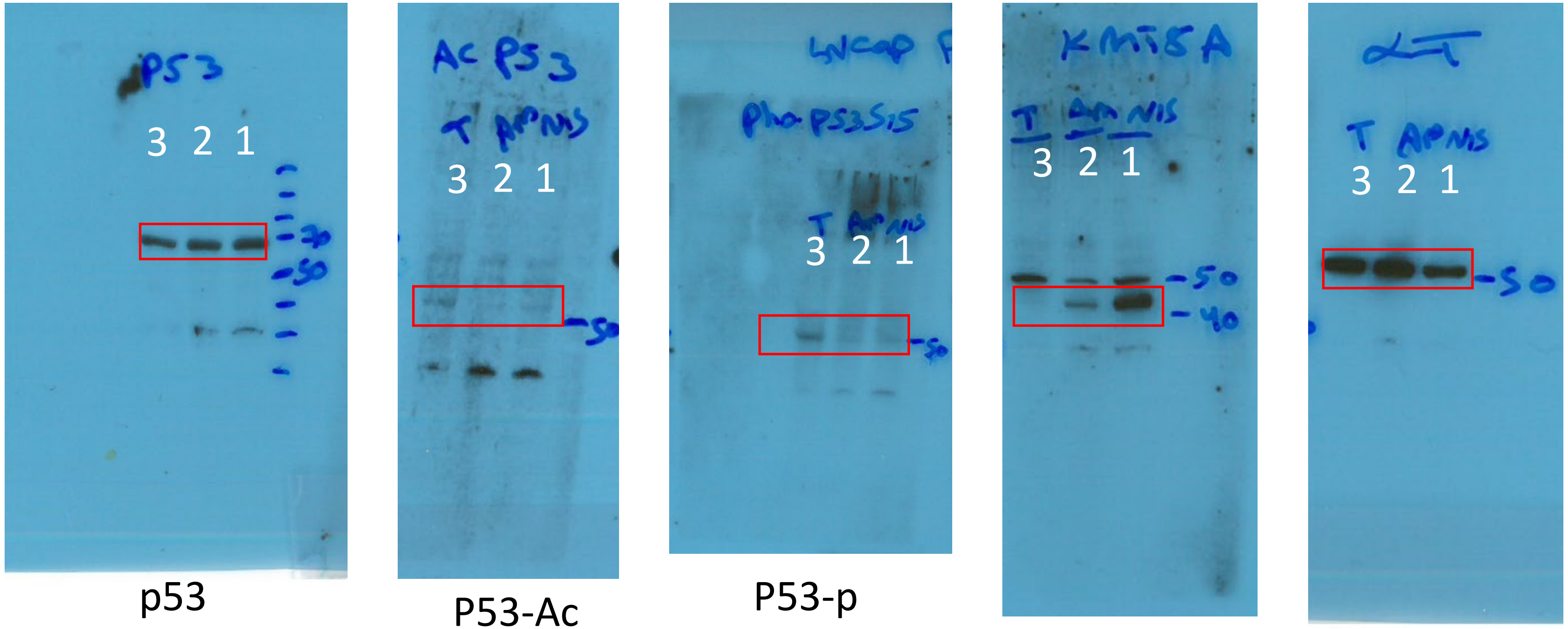

p53   P53-Ac   P53-p   KMT5A   Tubulin

1. N/S
2. siKMT5A_1
3. siKMT5A_2

LNCaP uncut blot experimental repeat 1

ZA
